# BlendSplice: A Frequency-Blended Generative Framework for *In Silico* Synthesis of Biologically Realistic Splice Site Sequences

**DOI:** 10.34133/csbj.0171

**Published:** 2026-07-23

**Authors:** Espoir Kabanga, Seonil Jee, Arnout Van Messem, Wesley De Neve

**Affiliations:** ^1^ IDLab, Department of Electronics and Information Systems, Ghent University, Ghent 9000, Belgium.; ^2^ Center for Biosystems and Biotech Data Science, Ghent University Global Campus, Incheon 21985, Republic of Korea.; ^3^ Department of Mathematics, Université de Liège, Liège 4000, Belgium.

## Abstract

Generative models for biological sequences face challenges in balancing sequence realism with diversity. We investigated whether posttraining frequency blending, combining model-learned distributions with empirical nucleotide priors, can improve synthetic-sequence quality across diverse generative architectures. We present BlendSplice, a frequency-blended generative framework for the *in silico* synthesis of biologically realistic splice site sequences. Our approach combines position-specific empirical nucleotide priors with 3 generative architectures: a one-dimensional U-Net-based denoising diffusion probabilistic model, a generative adversarial network, and a variational autoencoder. By linearly blending model-learned distributions with empirical base frequencies through a tunable weight parameter lambda (λ), we enable explicit control over the realism–diversity trade-off. We evaluate synthetic donor (5′) and acceptor (3′) splice sites from *Arabidopsis thaliana*, *Homo sapiens*, and *Danio rerio*, at 2 sequence lengths (402 and 2,002 bp), at λ=0.0 (no blending) and λ=0.5 (balanced blending of priors and model probabilities) using direct assessments (GC content, nucleotide conservation, sequence logos, k-mer divergence, and polypyrimidine tract analysis) and indirect functional validation via state-of-the-art splice site classifiers (SpliceRover and Spliceator) and independent validation with SpliceAI, under 3 evaluation protocols: Train-Real/Test-Synthetic (biological realism), Train-Synthetic/Test-Real (transferability), and fixed-budget data augmentation with varying real/synthetic mixtures. Our results demonstrate that frequency blending at λ=0.5 substantially improves biological fidelity and downstream predictive performance for the generative adversarial network and diffusion models, whereas the variational autoencoder model generates high-quality sequences even without blending. Data augmentation with a 50% real and 50% synthetic mix achieves predictive performance comparable to the baseline across all 3 species, indicating that blending is an effective strategy for generating high-quality synthetic genomic data.

## Introduction

RNA splicing is a critical eukaryotic process in which introns are removed from pre-messenger RNA (pre-mRNA) and exons are joined to form mature mRNA [1,2]. Accurate identification of donor (5′) and acceptor (3′) splice sites is essential for understanding gene structure, regulation, and alternative splicing mechanisms. Donor sites typically feature a GT dinucleotide at the exon–intron boundary, and acceptor sites display an AG motif at the intron–exon boundary [3,4], both surrounded by conserved sequence patterns that vary across species and influence spliceosome recognition [5,6].

Generating synthetic DNA sequences with authentic splice site characteristics addresses multiple research needs: augmenting limited datasets for machine learning [7], reducing reliance on direct sharing of sensitive patient genomic sequences through the use of synthetic genomes generated by generative neural networks [8], testing splice site prediction algorithms [9], and supporting comparative genomic studies [10]. Accurate splice site prediction further supports downstream applications such as the design of splice-modulating antisense oligonucleotides [11]. Traditional computational approaches, including position weight matrices [12], Markov models [13,14], and maximal dependence decomposition [15], rely primarily on local nucleotide dependencies as their complete generative mechanism, limiting their ability to capture long-range dependencies, combinatorial motif interactions, and higher-order sequence constraints that characterize functional splice sites [16]. Such local nucleotide dependencies also underlie many classical sequence representations, including k-mer-based approaches [17].

Recent advances in deep learning have enabled more sophisticated sequence generation. Recurrent neural networks and long short-term memory networks have been applied to DNA sequence modeling [18,19], capturing sequential dependencies but often struggling with long-range patterns. Generative adversarial networks (GANs) [20] have shown success in generating regulatory sequences [18,19], promoters [21], and enhancers [22], leveraging adversarial training to produce realistic samples. However, GANs face challenges including mode collapse, training instability, and difficulty generating discrete sequences. Variational autoencoders (VAEs) offer an alternative approach, learning continuous latent representations of sequences and enabling controllable generation [23]. VAEs have been successfully applied to protein sequence generation [24,25], regulatory element design [19], and DNA sequence modeling [26], providing stable training and interpretable latent spaces.

More recently, denoising diffusion probabilistic models have emerged as a powerful framework for high-quality sample generation [27,28]. Diffusion models iteratively refine random noise into structured data through a learned reverse diffusion process, demonstrating state-of-the-art performance in image generation [29,30], and have subsequently been extended to biological sequences.

DNA-Diffusion [31] introduced diffusion models for unconditional DNA sequence generation, demonstrating the ability to capture nucleotide distributions and short-range dependencies. Subsequent work extended diffusion models to discrete biological sequences [32,33], protein sequence generation [34,35], and biologically constrained optimization [36,37], highlighting their versatility across genomic and molecular design tasks. Transformer-based models have likewise been applied to nucleotide-sequence analysis [38], with recent studies examining the influence of tokenization, architecture, and alternative sequence models on genomic prediction performance [39,40].

Despite these advances, existing generative approaches for splice site sequences do not systematically incorporate position-specific nucleotide constraints that define splice site identity. Splice site recognition depends on precise positional information: invariant dinucleotides at junctions, purine enrichment upstream of donors for U1 small nuclear ribonucleoprotein (snRNP) binding [41], polypyrimidine tracts (PPTs) upstream of acceptors for U2AF recognition [42], and branch point sequences [43]. Standard generative models may learn these patterns implicitly, but the extent to which positional constraints are enforced varies substantially by architecture and training objective. We hypothesize that guiding generative models with empirical position-specific base frequencies can improve biological fidelity in architectures prone to compositional bias while remaining harmless for architectures that naturally capture positional structure and without interfering with each model’s capacity to learn higher-order dependencies.

**Fig. 1.**
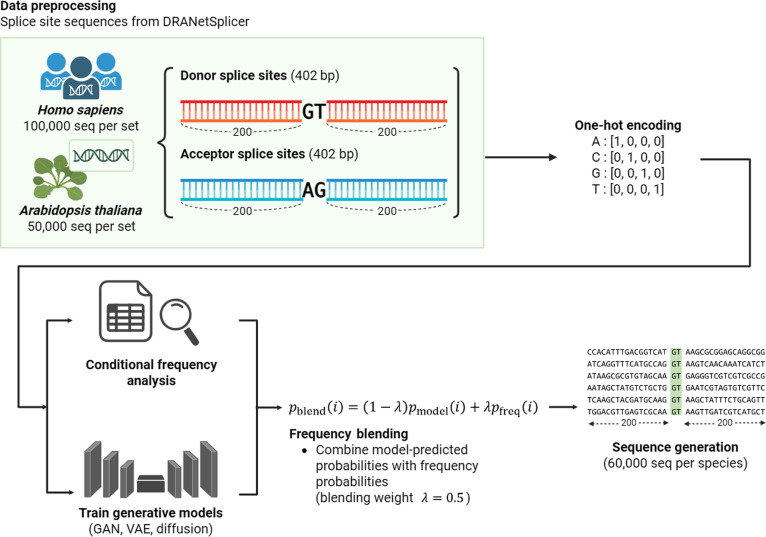
Three-stage model pipeline showing (a) data preprocessing of real positive sequences; (b) conditional frequency analysis and training of the generative adversarial network (GAN), variational autoencoder (VAE), and diffusion models; and (c) sequence generation.

We introduce BlendSplice, a frequency-blended generative framework that combines position-specific nucleotide priors with 3 generative models: a GAN, a VAE, and a diffusion model. Unlike approaches that incorporate biological constraints during training, BlendSplice operates only at sampling time by blending model-predicted nucleotide probabilities with empirical position-specific frequencies. This lightweight, posttraining, model-agnostic design preserves the generative model’s role in learning higher-order sequence structure while reinforcing well-established local splice site constraints. By linearly blending empirical base frequencies with model-learned probability distributions through a tunable parameter λ, we enable explicit control over the realism–diversity trade-off across 3 families of generative models. Our contributions are as follows: (a) a model-agnostic frequency-blending mechanism applicable to diverse generative architectures; (b) a comprehensive evaluation across 3 species (*Arabidopsis thaliana*, *Homo sapiens*, and *Danio rerio*) and 2 sequence lengths (402 and 2,002 bp), using complementary direct biological assessments (GC content, nucleotide conservation, sequence logos, k-mer divergence, and PPT quantification) and indirect functional validation with SpliceRover [44], Spliceator [45], and independent validation using SpliceAI [6]; and (c) an empirical demonstration that blending at λ=0.5 consistently improves biological fidelity and functional utility compared to unblended generation (λ=0.0) for GAN and diffusion models, but not necessarily for VAE models; and (d) the use of data augmentation with 50% real sequences as a practical strategy achieving predictive performance comparable to that of training on real sequences only. Figure 1 illustrates our 3-stage pipeline: (a) data preprocessing of real positive splice site sequences; (b) extraction of position-specific nucleotide frequencies and training of GAN, VAE, and diffusion models; and (c) synthetic-sequence generation where frequency blending is applied after model training by linearly combining model-learned probabilities with empirical priors using a tunable weight λ.

## Materials and Methods

### Datasets and preprocessing

We used the DRANetSplicer [46] dataset, which contains approximately 64,000 sequences for *A. thaliana* and approximately 120,000 sequences for *H. sapiens*, providing a positive and a negative set for each species. Each sequence is 402 bp long and centered on the annotated splice site position. Specifically, both the positive and negative sets are centered on GT (donor) or AG (acceptor) dinucleotides annotated as candidate splice sites; the positive set corresponds to verified splice sites of the specified donor or acceptor category, whereas the negative set comprises decoy GT/AG instances that are not true splice sites. For training the generative models, we randomly sampled 50,000 sequences from the positive sets for *A. thaliana* and 100,000 sequences from the positive sets for *H. sapiens* per splice category (donor and acceptor) while reserving remaining sequences for evaluation to prevent data leakage. Sequences were encoded as 4-channel one-hot tensors over A, C, G, and T.

To test generalization to a broader sequence context and additional species, we constructed an independent extended-context dataset from primary genome assemblies (Ensembl/GENCODE) for *A. thaliana* (TAIR10), *H. sapiens* (GRCh38, GENCODE v49), and *D. rerio* (GRCz11). For each exon–intron boundary, we extracted a 2,002-bp window (1,000 bp + GT/AG + 1,000 bp), reverse-complementing minus-strand sequences so the splice site is centered, with matched intronic GT/AG decoys as negatives. After deduplicating sites by genomic coordinates, this yielded 63,115 (donor) and 67,231 (acceptor) positive sites for *A. thaliana*, 117,666 (donor) and 130,049 (acceptor) for *D. rerio*, and 174,505 (donor) and 185,375 (acceptor) for *H. sapiens*, each with an equal number of matched negatives. To separate context length from data source for *D. rerio*, for which no 402-bp benchmark dataset was available, we also derived 402-bp windows from the same extended-context pipeline by symmetric trimming, so that the 2 settings differ only in length. We sampled 50,000 positive and 50,000 negative sequences per species and category for training, reserving the rest for evaluation, with identical one-hot encoding.

### Conditional frequency analysis

To guide the generative models toward biologically realistic sequences, we computed conditional nucleotide frequency distributions from real splice site sequence alignments. These distributions represent the probability of observing a nucleotide at a given position conditioned on the nucleotide in the adjacent position, either upstream (previous neighbor) or downstream (next neighbor).

For each sequence $S=\left(s_{0},s_{1},\;\ldots ,s_{L-1}\right)$, where L denotes the sequence length (L=402 or L=2,002), with $s_{i}\in \left\{A,C,G,T\right\}$, we write

$$P\left(s_{i}|s_{i-1}\right),\;0<i\leq L-1;\;\;P\left(s_{i}|s_{i+1}\right),\;0\leq i<L-1,$$ (1)

for previous- and next-neighbor conditioning, respectively. The conditional probability of the previous neighbor is estimated as

$$P\left(s_{i}=x|s_{i-1}=b\right)=\frac{\text{Count}\left(s_{i-1}=b,s_{i}=x\right)}{\text{Count}\left(s_{i-1}=b\right)},$$ (2)

where $b,x\in \left\{A,C,G,T\right\}$. The next-neighbor probabilities are estimated analogously. At each interior position i, both the previous-neighbor distribution $P\left(s_{i}|s_{i-1}\right)$ and the next-neighbor distribution $P\left(s_{i}|s_{i+1}\right)$ are evaluated, and the 2 are averaged to form a single position-specific prior $p_{\text{freq}}\left(i\right)$ over $\left\{A,C,G,T\right\}$; at the sequence boundaries, where only one neighbor is defined, that single distribution is used. This prior $p_{\text{freq}}\left(i\right)$ is the empirical term that is combined with the model-predicted distribution in the blending step.

Table 1 shows an excerpt of a previous-neighbor conditional frequency table computed from real donor site sequences (*A. thaliana* dataset, positions 198 to 202). At position 200 in *A.* donor sites, when the previous nucleotide is A, the conditional probability $P\left(G|A\right)=0.880$, reflecting the strong conservation of the donor GT motif.

**Table 1.** Excerpt of previous-neighbor conditional frequencies for donor sequences (*Arabidopsis*); positions 198 to 202. For each position i and conditioning base b, $P\left(x|b\right)$ is shown for $x\in \left\{A,C,G,T\right\}$.

Pos.	Prev.	$P\left(A|b\right)$	$P\left(C|b\right)$	$P\left(G|b\right)$	$P\left(T|b\right)$
198	A	0.340	0.160	0.280	0.220
199	A	0.150	0.100	0.700	0.050
200	A	0.030	0.040	0.880	0.050
201	G	0.000	0.000	0.000	1.000
202	T	0.420	0.180	0.200	0.200

We use these position-specific nucleotide probabilities as priors at sampling time (sequence generation phase, after model training) to blend model outputs with empirical frequencies. Because blending is applied only during sampling, it preserves the generative model’s role in learning higher-order sequence structure while reinforcing well-established local splice site constraints.

### Generative models

We evaluated 3 generative architectures: a fully connected GAN, a convolutional VAE, and a one-dimensional U-Net [47] diffusion model. These cover adversarial, variational, and denoising modeling frameworks with inductive biases toward global mapping, local motif modeling, and multiscale refinement.

#### Generative adversarial network

Our GAN consists of 2 competing networks: a generator that maps 100-dimensional Gaussian noise through fully connected layers to produce $\left(L,4\right)$ sequence representations (where L denotes the sequence length and 4 denotes nucleotide channels) and a discriminator that classifies sequences as real or synthetic. We train both networks in alternating steps using the binary cross-entropy loss. The discriminator learns to distinguish real from generated sequences, while the generator learns to produce sequences that fool the discriminator. This adversarial process continues until the generator produces realistic sequences. To generate sequences, we sample noise vectors $z∼N\left(0,I_{100}\right)$ and pass them through the generator to obtain raw logits. We apply softmax normalization to convert these to position-wise nucleotide probability distributions over $\left\{A,C,G,T\right\}$. The resulting nucleotide probability distributions are subsequently processed using the frequency-blending procedure, where they are combined with empirical frequency priors before generating the final sequences. Detailed model specifications are provided in Table 2.

**Table 2.** GAN hyperparameters and architecture details

Parameter	Value/configuration
Generator	
Input dimension	100 (Gaussian noise $z∼N\left(0,I_{100}\right)$)
Hidden layers	64 → 128 → 256 → 512
Output dimension	4L (reshaped to $\left(L,4\right)$; 1,608 for L=402, 8,008 for L=2,002)
Activation function	LeakyReLU (α=0.2)
Normalization	Batch normalization
Dropout rate	0.3
Learning rate	5 × 10^−5^
Discriminator	
Input dimension	4L (flattened from $\left(L,4\right)$; 1,608 for L=402, 8,008 for L=2,002)
Hidden layers	512 → 256 → 128
Output dimension	1 (binary classification)
Activation function	ReLU (hidden), sigmoid (output)
Learning rate	2 × 10^−4^
Training configuration	
Loss function	Binary cross-entropy
Optimizer	Adam
Batch size	512
Number of epochs	50
Inference	
Noise sampling	100-dimensional standard Gaussian: $z∼N\left(0,I_{100}\right)$
Output processing	Softmax normalization + multinomial sampling

GAN, generative adversarial network

#### Variational autoencoder

Our VAE architecture, inspired by DiscDiff [32], uses a convolutional encoder to compress sequences into a 128-dimensional latent space and a transposed convolutional decoder to reconstruct sequences from latent representations. The encoder and decoder architectures follow the VAE framework [23]. We optimize the evidence lower bound, combining cross-entropy reconstruction loss with Kullback–Leibler divergence regularization. This encourages the model to learn a structured latent space where similar sequences cluster together while maintaining compatibility with a standard Gaussian prior $N\left(0,I_{128}\right)$. To generate sequences, we sample latent vectors $z∼N\left(0,I_{128}\right)$ from the prior and decode them through the decoder network to obtain raw logits of size $\left(L,4\right)$. We apply softmax normalization to convert these to position-wise nucleotide probability distributions. For frequency blending, we linearly combine the model-predicted probabilities at each position with conditional empirical frequency priors computed from neighboring nucleotides in real training sequences. These frequency priors incorporate both previous- and next-neighbor conditioning to capture local sequence context. After blending with weight λ, we enforce splice site constraints and sample discrete nucleotides at each position from the resulting categorical distribution. Detailed model specifications are provided in Table 3.

**Table 3.** VAE hyperparameters and architecture details

Parameter	Value/configuration
Encoder	
Input shape	$\left(4,L\right)$ one-hot encoded sequence
Conv1D layers	3 layers: 4 → 32 → 64 → 128 channels
Kernel sizes	5, 5, 3
Pooling	Max-pooling (kernel size 2) after each layer
Spatial reduction	402→201→100→50 (L=402); 2,002→1,001→500→250 (L=2,002)
Flattened dimension	128×50=6,400 (L=402); 128×250=32,000 (L=2,002)
Latent dimension	128 (μ and $\log\sigma^{2}$)
Activation function	ReLU
Decoder	
Input dimension	128 (latent vector z)
Linear projection	$128\to \left(128,50\right)$ (L=402); $128\to \left(128,250\right)$ (L=2,002)
TransposeConv1D layers	4 layers: 128 → 64 → 32 → 16 → 4
Output shape	$\left(4,L\right)$ reconstructed sequence
Activation function	ReLU
Training configuration	
Loss function	ELBO (reconstruction + KL divergence)
Reconstruction loss	Cross-entropy
Optimizer	Adam
Learning rate	10^−3^
Batch size	32
Number of epochs	50
Inference	
Latent sampling	$z∼N\left(0,I_{128}\right)$ from prior
Output processing	Softmax normalization + multinomial sampling

VAE, variational autoencoder; ELBO, evidence lower bound; KL, Kullback–Leibler

#### Diffusion model

Our diffusion model, inspired by DNA-Diffusion [31], employs a one-dimensional U-Net architecture [47] with time-step conditioning to predict noise at each step of a 50-step diffusion process following the denoising diffusion framework [27]. We train the model to predict noise added to sequences at arbitrary time steps using the mean squared error loss. At each training iteration, we corrupt sequences by progressively adding Gaussian noise in a linear way: the noise level $\beta_{t}$ increases linearly from 0.0001 to 0.02 over T=50 diffusion time steps. U-Net learns to predict the added noise conditioned on the current time step. Generation begins with pure Gaussian noise $z_{T}∼N\left(0,I_{4L}\right)$ and proceeds through 50 iterative denoising steps. At each step t, the model predicts the noise component and removes it to obtain $z_{t-1}$. After the final denoising step, we obtain raw logits of size $\left(L,4\right)$ and apply softmax normalization to convert these to position-wise nucleotide probability distributions. For frequency blending, we linearly combine the model probabilities with conditional empirical frequency priors that depend on neighboring nucleotides. The frequency priors are computed iteratively based on the current sequence state, using both previous- and next-neighbor conditioning from conditional frequencies extracted from the training data. After blending with weight λ, we enforce splice site constraints and sample discrete nucleotides at each position from the resulting categorical distribution. Detailed model specifications are provided in Table 4.

**Table 4.** Diffusion model hyperparameters and architecture details

Parameter	Value/configuration
U-Net architecture	
Input shape	$\left(4,L\right)$ noisy sequence at time t
Encoder channels	32 → 64 → 128 → 256
Convolution blocks	Double Conv1D-BatchNorm-ReLU per stage
Pooling	Max-pooling (kernel size 2)
Spatial reduction	402→201→100→50 (L=402); 2,002→1,001→500→250 (L=2,002) (bottleneck)
Time embedding network	2-layer multilayer perceptron (MLP): 1 → 128 → 256
Time embedding activation	SiLU (Swish)
Decoder channels	256 → 128 → 64 → 32 → 4
Skip connections	Between corresponding encoder–decoder layers
Output shape	$\left(4,L\right)$ predicted noise $\epsilon_{\theta }$
Diffusion process	
Number of diffusion steps	50 (T=50)
Noise schedule	Linear: $\beta_{t}\in \left[0.0001,0.02\right]$
Forward process	$q\left(z_{t}|z_{t-1}\right)=N\left(\sqrt{1-\beta_{t}}z_{t-1},\beta_{t}I\right)$
Training configuration	
Loss function	Mean squared error (MSE)
Objective	${\left\|\epsilon -\epsilon_{\theta }\left(z_{t},t\right)\right\|}^{2}$
Optimizer	Adam
Learning rate	10^−4^
Batch size	64
Number of epochs	50
Inference	
Initialization	$z_{T}∼N\left(0,I_{4L}\right)$ (pure noise; $I_{1,608}$ for L=402, $I_{8,008}$ for L=2, 002), with T=50
Reverse steps	50 iterative denoising steps
Output processing	Softmax normalization + argmax decoding

### Blending weight and sequence generation

Blending is applied only during sequence generation and leaves training untouched. Each generative model (GAN, VAE, and the diffusion model) independently produces position-wise logits over $\left\{A,C,G,T\right\}$, which we convert to probabilities using a row-wise softmax. For a sequence of length 402, let $i\in \left\{0,1,\;\ldots ,401\right\}$ denote the position index, and let $p_{\text{model}}\left(i\right)\in {\mathbb{R}}^{4}$ denote the probability vector obtained for position i for a given model. Let $p_{\text{freq}}\left(i\right)\in {\mathbb{R}}^{4}$ be the context-specific prior computed from conditional nucleotide frequencies as defined in Eq. (2). We form

$$p_{\text{blend}}\left(i\right)=\left(1-\lambda \right)\;p_{\text{model}}\left(i\right)+\lambda \;p_{\text{freq}}\left(i\right),$$ (3)

with $\lambda \in \left\{0,0.25,0.5,0.75\right\}$. The choice of λ=0 corresponds to the model predictions without frequency blending, which will be used as a baseline. The values λ=0.25 (more weight to model-learned probabilities) and λ=0.75 (more weight to frequency prior probabilities) represent different trade-offs between model-learned patterns and empirical priors. These 4 values, $\lambda \in \left\{0,0.25,0.5,0.75\right\}$, are evenly spaced and span the range from pure model predictions (λ=0) toward an increasing contribution of the empirical priors, with λ=0.5 as the balanced midpoint that weights model predictions and empirical priors equally. Through systematic evaluation of the biologically relevant quality metrics GC content, nucleotide conservation scores, sequence logos, and downstream splice site classifiers’ performance, we selected λ=0.5 as the optimal value that balances the influence of model predictions and empirical frequencies while preventing either from dominating the generation process. The nucleotide at each position $i\in \left\{0,1,\;\ldots ,L-1\right\}$, where L denotes the sequence length (L=402 or L=2,002), is then selected from the blended probability vector $p_{\text{blend}}\left(i\right)$ as

$$s_{i}=\left\{\begin{array}{cc} \text{arg}\underset{k\in \left\{A,C,G,T\right\}}{\text{max}}p_{\text{blend}}{\left(i\right)}_{k}, & \text{diffusion},\\ ∼\text{Categorical}\;\left(p_{\text{blend}}\left(i\right)\right), & \text{GAN}\;\text{and}\;\text{VAE}, \end{array}\right.$$ (4)

where $p_{\text{blend}}\left(i\right)$ is obtained by softmax normalization of the model output, optional λ-weighted blending with neighbor-conditional frequency priors, and enforcement of the canonical splice site motif at the central positions.

Table 5 shows a hypothetical illustrative example at an arbitrary position i.

**Table 5.** Example of nucleotide probability blending at position i with λ=0.5.

Base	Model	Prior	Blended
A	0.100	0.200	0.150
C	0.150	0.300	0.225
G	0.600	0.400	0.500
T	0.150	0.100	0.125

In this example

• The model predicts G with high confidence (0.60), while empirical frequency data show C and G with probabilities 0.30 and 0.40, respectively.
• Blending reduces overconfidence in G and slightly increases the probabilities for C, A, and T.
• The final nucleotide is selected as the one with the highest blended probability (here, G with probability 0.50).

Using this procedure with the optimal blending weight λ=0.5, we generated, for each species (*A. thaliana*, *H. sapiens*, and *D. rerio*) and both splice categories (donor and acceptor), 60,000 synthetic sequences at 402 bp and 20,000 at the extended context of 2,002 bp. These quantities were chosen so that the synthetic sets are comparable in scale to the real datasets, providing a sufficiently large sample for stable estimation of the evaluation metrics. The smaller count for the 2,002-bp sequences reflects the substantially higher cost of generating longer sequences; a generation-time analysis is provided in the Supplementary Materials.

## Evaluation Strategy

We adopt 2 complementary evaluation strategies: (a) a direct evaluation strategy that assesses the fidelity (realism) of synthetic sequences using descriptive sequence statistics and motif-level structure and (b) an indirect evaluation strategy via proxy prediction tasks with splice site detectors. Both evaluations are conducted separately for each species and splice category and are reported for 3 data conditions: Real (real sequences), No-Blend (synthetic sequences generated without frequency blending, λ=0), and Blend (synthetic sequences generated with frequency blending, λ=0.5). To assess the sensitivity of our results to the blending weight and whether the chosen value generalizes across datasets and architectures, we additionally evaluate sequences generated across a range of λ values ($\lambda \in \left\{0,0.25,0.5,0.75\right\}$). The full sensitivity analysis is reported in the Supplementary Materials.

### Direct evaluation

For the direct evaluation, we first examine GC content by computing the percentage of G or C bases in each sequence and comparing the resulting distributions and means across the 3 data conditions; close correspondence indicates preservation of global base composition. In addition to comparing means, we summarize each distribution using box plots (interquartile range [IQR], whiskers, and mean) so that differences in distributional shape, and not only in central tendency, are captured. Next, we derive nucleotide conservation scores by computing, at each position, the proportion of sequences carrying the most frequent nucleotide; this position-wise curve reflects motif sharpness and positional specificity. To localize differences relative to Real, we additionally render these scores as heatmaps of Δconservation (synthetic − Real) over a ±50-bp window around the splice site. Finally, we estimate position weight matrices from empirical nucleotide frequencies and render sequence logos within a focused window around the junction, enabling a visual comparison of motif structure and base preferences across Real, No-Blend, and Blend sequences.

To complement these descriptive analyses with quantitative measures, we compute 2 additional statistics. First, we quantify distributional similarity using the Jensen–Shannon divergence (JSD) between the k-mer frequency spectra of real and synthetic sequences, for k=3 and k=6; the JSD is bounded in $\left[0,1\right]$, with lower values indicating closer agreement with Real, and is evaluated separately for each species, splice category, and sequence length on matched sample sizes. Second, because acceptor recognition depends on the PPT, we score this element in acceptor sequences over positions −40 to −5 relative to the AG. We report tract severity as the mean pyrimidine (C/T) fraction in this window, tract integrity as the mean longest consecutive pyrimidine run, and malformation frequency as the fraction of sequences whose pyrimidine fraction falls below the 5th percentile of Real, with the threshold determined separately for each species and sequence length.

### Indirect evaluation

For the indirect evaluation, we assess the functional realism of our synthetic sequences using 2 state-of-the-art splice site prediction architectures: SpliceRover [44] and Spliceator [45]. These models were selected based on their demonstrated high effectiveness in splice site prediction. The detailed architectures and training setup for both models are provided in the Supplementary Materials. The SpliceRover architecture is defined separately for each input sequence length, with the network depth increasing with length, and is specified only for sequences of up to 400 bp; it therefore could not be applied to the extended-context 2,002-bp sequences. Spliceator, in contrast, uses a single fixed architecture that accommodates arbitrary input lengths and was applied successfully at both lengths. We therefore use both SpliceRover and Spliceator as proxy classifiers for the 402-bp sequences and Spliceator alone for the 2,002-bp sequences.

To provide validation that is independent of these 2 classifiers, we additionally evaluate our synthetic sequences using SpliceAI [6], a widely used splice site predictor with a different architecture and trained on a different dataset. We use the publicly available pretrained SpliceAI model without any retraining, so that it serves as a fully external validator. Because our generated sequences have the splice site at a fixed central position, we apply SpliceAI to each sequence and record its predicted score at that position for the corresponding site type (donor or acceptor). As our evaluation measure, compared against real sequences as the reference, we report the detection rate, defined as the fraction of sequences for which SpliceAI’s score at the junction exceeds a threshold (τ=0.5). This independent evaluation is applied to both the 402-bp and the extended-context 2,002-bp sequences, complementing the proxy classifiers and providing long-context validation where SpliceRover cannot be used.

We evaluate synthetic-sequence quality under 3 complementary scenarios, using models trained and tested on real sequences (Train-Real/Test-Real) as the baseline for comparison:

Scenario 1: Train-Real/Test-Synthetic (biological realism). We train splice site classifiers exclusively on real sequences and test on synthetic sequences generated by each model (GAN, VAE, and diffusion) with and without blending. This scenario quantifies how well synthetic sequences preserve the discriminative patterns learned from real sequences. High predictive performance indicates that synthetic sequences maintain the biological features necessary for splice site recognition.

Scenario 2: Train-Synthetic/Test-Real (transferability). We train classifiers exclusively on synthetic sequences and test on the remaining real sequences. We use synthetic sequences generated at λ=0.5 as it equally balances the empirical frequency priors and the model-learned predictions. This scenario quantifies the domain shift between synthetic- and real-sequence distributions and establishes whether blending enhances model generalization and transferability to real genomic sequences.

Scenario 3: Data augmentation. We train classifiers on mixtures of real and synthetic sequences at varying proportions (10%, 25%, and 50% real sequences, with the remainder synthetic, and with λ=0.5), keeping the total training set size fixed. Models are evaluated on real test sequences to determine whether augmenting limited real training data with synthetic sequences improves performance compared to training on real sequences alone. In all scenarios, the training set size is fixed at 50,000 sequences per species and splice category, with matched class balance and identical preprocessing and hyperparameters. Evaluation for the baseline, scenario 2, and scenario 3 was performed on a held-out real test set drawn from the EnsembleSplice dataset [48]. Test sequences were 602 bp with the splice site centered; we trimmed symmetrically to 402 bp so the splice site remains centered, matching the length and format of the training sequences. Across all scenarios, the predictive performance of the classifiers is measured using 3 complementary metrics, each capturing a different aspect of predictive quality.

The F1-score provides the harmonic mean of precision and recall, offering a balanced measure when class distributions may be imbalanced:

$$F1\;\text{-score}=2\times \frac{\text{Precision}\times \text{Recall}}{\text{Precision}+\text{Recall}}=\frac{2\text{TP}}{2\text{TP}+\text{FP}+\text{FN}}.$$ (5)

The Matthews correlation coefficient (MCC) quantifies the correlation between predicted and actual classifications, with values ranging from −1 (complete disagreement) to +1 (perfect prediction):

$$\text{MCC}=\frac{\text{TP}\times \text{TN}-\text{FP}\times \text{FN}}{\sqrt{\left(\text{TP}+\text{FP}\right)\left(\text{TP}+\text{FN}\right)\left(\text{TN}+\text{FP}\right)\left(\text{TN}+\text{FN}\right)}}.$$ (6)

MCC is particularly robust to class imbalance and provides a single value summary of the confusion matrix. TP, TN, FP, and FN denote true positives, true negatives, false positives, and false negatives, respectively.

The area under the receiver operating characteristic curve (AUROC) evaluates discriminator performance across all classification thresholds, measuring the probability that the model ranks a randomly chosen real sequence higher than a randomly chosen generated sequence. AUROC values range from 0.5 (random classification) to 1.0 (perfect discrimination).

## Results

### Direct evaluation

**Fig. 2.**
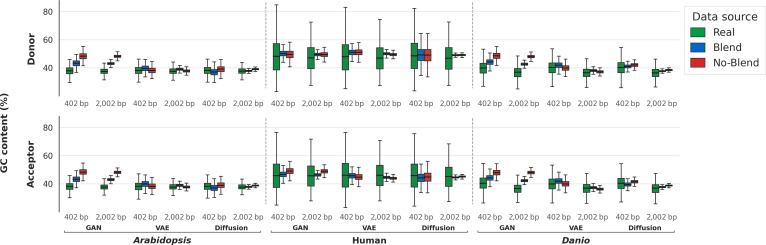
GC content at donor (top) and acceptor (bottom) splice sites for real sequences and synthetic sequences from the generative adversarial network (GAN), variational autoencoder (VAE), and diffusion models, evaluated in *Arabidopsis*, human, and *Danio* at 402- and 2,002-bp context lengths. Green, blue, and red boxes denote Real, Blend (λ=0.5), and No-Blend (λ=0) sequences, respectively. Horizontal lines mark the mean; donor and acceptor panels share the same y-axis.

Figure 2 compares GC content distributions using box plots for donor (top) and acceptor (bottom) splice sites in *A. thaliana*, *H. sapiens*, and *D. rerio*. For each species, generative model (GAN, VAE, and diffusion), and context length (402 and 2,002 bp), 3 box plots show Real, Blend (λ=0.5), and No-Blend (λ=0) sequences. Boxes indicate the IQR, whiskers extend to 1.5×IQR, and horizontal lines denote the mean. For *H. sapiens*, the Real distribution is centered near 46% to 48% GC (donor mean 48.42%, standard deviation [SD] 11.80% at 402 bp; 46.75%, SD 9.54% at 2,002 bp) but is broad, whereas the synthetic distributions are centered at comparable means with much smaller SDs (SD ≈ 2.5% to 5.6% at 402 bp). The central tendency of Real and synthetic sequences is therefore similar, while their spread is not; the analysis below focuses on this difference in distributional shape rather than on mean values alone.

For GAN-generated sequences, the box plots reveal substantial GC inflation in *A. thaliana* at both window sizes. At 402 bp, the No-Blend donor mean reached 48.28% versus 38.15% for Real (a difference of about 10%), with Blend reducing the mean to 43.32%; at 2,002 bp, the corresponding values were 48.16%, 37.51%, and 43.04%. Acceptor sites showed the same pattern (402 bp: 48.14% versus 38.20%; 2,002 bp: 48.10% versus 37.69%). *D. rerio* exhibited a similar GAN bias (402-bp donor: 48.38% versus 40.18%; 2,002-bp donor: 48.02% versus 36.66%). In *H. sapiens*, the GAN means were closer to Real (402-bp donor: 49.53% for No-Blend and 50.25% for Blend versus 48.42% for Real), but the Real sequences were far more variable (donor SD 11.80% at 402 bp) than the GAN sequences (SD 3.31% and 2.62%, respectively). Mean similarity therefore coexisted with a clear difference in spread.

For VAE-generated sequences, the No-Blend means closely matched Real GC in *A. thaliana* at both context lengths (402-bp donor: 38.28% versus 38.14%; 2,002-bp donor: 37.79% versus 37.53%), with Blend introducing modest inflation (about 1.5% at both lengths). *D. rerio* No-Blend means were also near Real values (402-bp donor: 40.12% versus 40.24%). In *H. sapiens*, behavior depended on splice site type: donor means lay above Real at both lengths (402 bp No-Blend/Blend: 50.99%/50.96% versus 48.50%; 2,002 bp: 49.46%/50.06% versus 46.77%), whereas acceptor means fell slightly below Real (402 bp: 44.62%/45.62% versus 46.29%; 2,002 bp: 43.94%/44.65% versus 45.46%). As with GAN, the VAE sequences were much less variable than Real (human donor SD 2.48% for No-Blend versus 11.91% for Real at 402 bp).

For diffusion-generated sequences, the *A. thaliana* means remained within about 1% of Real at both context lengths (402-bp donor No-Blend: 39.09% versus 38.09%; 2,002 bp: 39.04% versus 37.51%). In *H. sapiens*, donor means were slightly above Real at both lengths (402 bp No-Blend/Blend: 49.11%/49.12% versus 48.37%; 2,002 bp: 49.05%/48.99% versus 46.72%), while the acceptor Blend mean showed the largest deficit at 402 bp (43.96% versus 46.13%) and a smaller deficit at 2,002 bp (44.50% versus 45.55%). The diffusion sequences were less variable than Real at 402 bp (human donor SD 5.62% versus 11.77%) and tighter still at 2,002 bp (SD 0.61%), indicating stronger concentration around the conditional mean at longer context.

Across all models, all species, and both context lengths, the synthetic sequences were consistently less variable than the Real sequences, indicating that similarity in mean GC does not imply similarity in distributional shape. For *H. sapiens* in particular, the Real distributions retained wide IQRs and long whiskers (donor SD around 9% to 12%), whereas the generated sequences formed compact boxes (SD around 1% to 6%) near 44% to 51% GC. Because GC content affects pre-mRNA secondary structure and splice site recognition [49], these differences in spread, together with model-specific mean biases (GAN inflation in *A. thaliana* and *D. rerio*, VAE donor elevation and acceptor shortfall in *H. sapiens*, and diffusion acceptor deficits), indicate an incomplete capture of GC diversity. We therefore report this as a current limitation of the generators rather than a property addressed by blending.

**Fig. 3.**
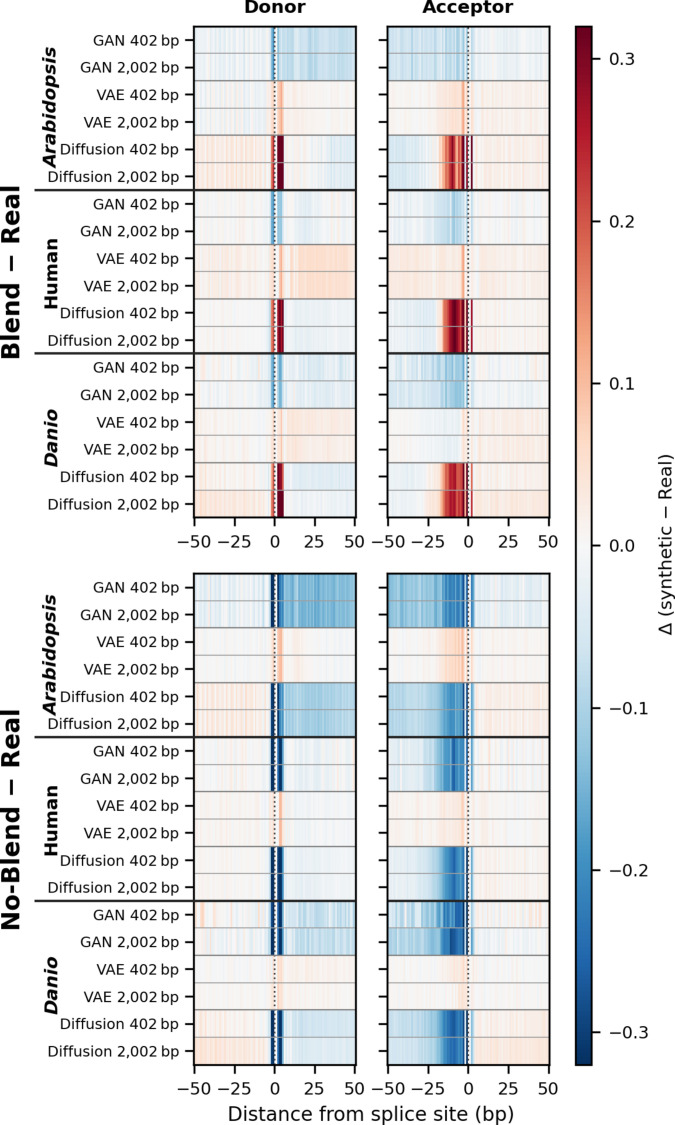
Nucleotide conservation fidelity at donor and acceptor splice sites, shown as the difference between synthetic and real sequences (Blend − Real and No-Blend − Real) for the generative adversarial network (GAN), variational autoencoder (VAE), and diffusion models in *Arabidopsis*, human, and *Danio*, at 402- and 2,002-bp context lengths. Each heatmap row corresponds to one species–model–context condition; the x-axis spans ±50 bp relative to the splice site. Red and blue indicate higher and lower conservation in synthetic sequences relative to real sequences, respectively. Horizontal dividers separate species, generative models, and context lengths.

Figure 3 summarizes nucleotide conservation fidelity as heatmaps of Δconservation (synthetic − Real) over ±50 bp relative to the splice site, separately for donor and acceptor sites, Blend (λ=0.5), and No-Blend (λ=0) sequences. Each row corresponds to one species–model–context (402 or 2,002 bp) condition; blue indicates lower conservation in synthetic sequences than in Real, and red indicates higher conservation. Patterns were qualitatively similar at 402 and 2,002 bp.

For the GAN model, the heatmaps were predominantly blue, indicating systematic underconservation relative to Real. At *A. thaliana* donors, No-Blend showed the strongest deficits immediately downstream of the splice site, whereas Blend reduced the magnitude of these negative deltas and tracked Real more closely across the downstream window. A similar pattern appeared at *A. thaliana* acceptors: No-Blend displayed broad negative Δconservation upstream, while Blend partially restored conservation toward Real in that region. In *H. sapiens* donors, downstream Δconservation was near zero for both variants, but Blend again showed smaller negative deviations at the splice site than No-Blend. In *H. sapiens* acceptors, Blend produced stronger positive Δconservation in the upstream intronic region than No-Blend, consistent with an oversharpening of the upstream conservation rise, whereas No-Blend remained closer to Real. *D. rerio* exhibited the same GAN trend: site-proximal blue bands for No-Blend, partially corrected by Blend.

By contrast, under the VAE model, the Δconservation heatmaps were much weaker and largely near zero, with both Blend and No-Blend closely matching Real across species and splice site types. Residual deviations were small and mostly positive (slight red tint) near the splice site and, for *H. sapiens* donors, in the downstream segment, where Blend showed marginally higher conservation than No-Blend. Blend introduced only minor adjustments relative to No-Blend, and no substantial species-specific failure mode was apparent.

For the diffusion model, the heatmaps revealed a consistent split between Blend and No-Blend at the splice site. *A. thaliana* donors showed red site-proximal Δconservation for Blend but blue deficits for No-Blend just downstream of the donor site, with Blend less negative than No-Blend in the downstream window. *H. sapiens* donors were well matched in the flanking regions, with both variants near zero away from the site. For *A. thaliana* acceptors, Blend reproduced the expected upstream increase in conservation (positive Δconservation upstream), whereas No-Blend remained below Real in the same region. In *H. sapiens* acceptors, Blend again produced stronger upstream and site-proximal positive Δconservation than No-Blend, indicating an oversharpening of the upstream rise, while No-Blend showed smaller deviations from Real. *D. rerio* followed the same diffusion model pattern as the other species.

Overall, the conservation heatmaps complement the GC box plots by localizing model errors in sequence space: GAN primarily reduces conservation relative to Real, especially under No-Blend; VAE preserves conservation with only minor deviations; and diffusion shows a Blend-dependent overshoot at the splice site, with No-Blend more often underconserved in the upstream regions of acceptor sites.

**Fig. 4.**
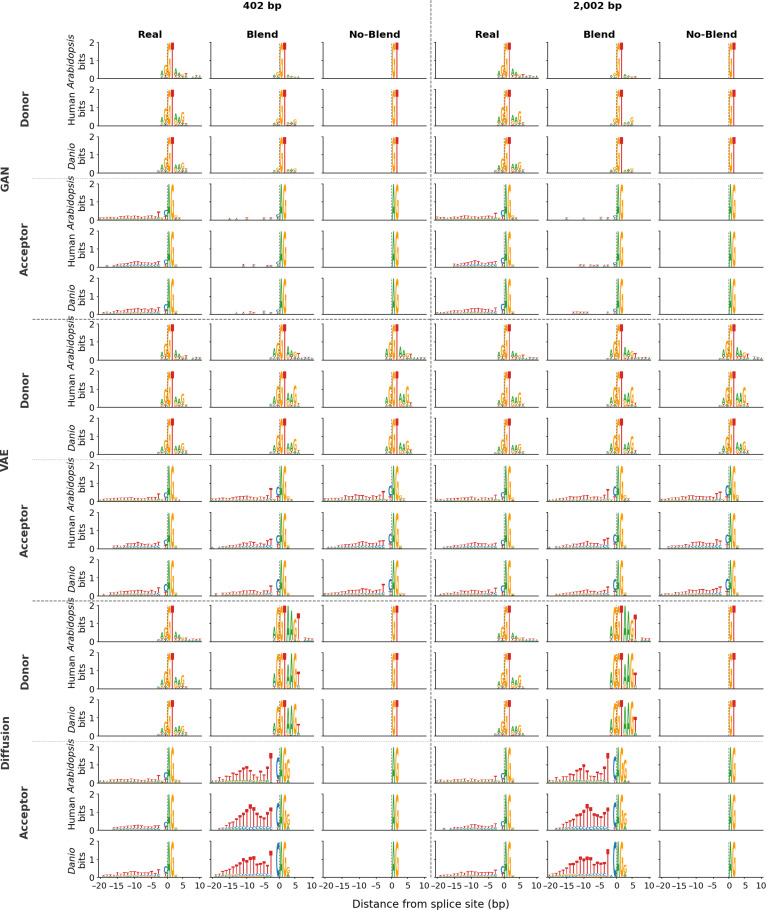
Sequence logos comparing generative adversarial network (GAN), variational autoencoder (VAE), and diffusion synthetic splice sites to real positives in *Arabidopsis thaliana*, human, and *Danio rerio*, for donor and acceptor sites in 402- and 2,002-bp contexts. Real, Blend (λ=0.5), and No-Blend (λ=0) sequences are shown side by side; information content (bits) is plotted from −20 to −1 (exon) and +1 to +10 (intron) relative to the splice junction.

Across models and species, sequence logos recover the canonical junction dinucleotides (GT for donors and AG for acceptors) in both 402- and 2,002-bp contexts (Fig. 4). In *A. thaliana* donors, Real sequence logos show a purine-rich segment immediately upstream of the splice site and an A-biased segment downstream; using the GAN model, the No-Blend sequence logo attenuates both features, whereas the Blend sequence logo moves toward the Real pattern but remains slightly flatter upstream. At *A. thaliana* acceptors, the Blend sequence logo also increases upstream T relative to No-Blend immediately before the splice site. In *H. sapiens*, for the GAN model, the Real acceptor shows a clear upstream PPT; the Blend sequence logo raises upstream C/T relative to No-Blend and aligns closely with Real immediately upstream, while donors show only minor differences among the 3 sequence logos. *D. rerio* follows the same broad trends: canonical junctions are preserved under GAN, with blending mainly improving upstream acceptor composition relative to No-Blend.

For the VAE model, the Real, Blend, and No-Blend logos closely match across species and splice types (purine enrichment upstream of donors, A-bias downstream of donors, and an upstream PPT at human acceptors), with only small adjustments under blending (qualitatively similar at 402 and 2,002 bp).

For the diffusion model, *A. thaliana* donors are well captured by both synthetic logos, with the Blend logo yielding only a slight sharpening at the junction; in *H. sapiens* acceptors, the Blend logo recovers upstream C/T enrichment but oversharpens the upstream rise relative to Real, whereas the No-Blend logo shows weaker upstream pyrimidine content. A similar improvement of upstream pyrimidine content under blending is seen for *D. rerio*, at both sequence lengths.

To quantify distributional similarity beyond the descriptive analyses above, we computed the JSD between the k-mer spectra of real and synthetic sequences for k=3 and k=6 (Table 6; lower values indicate closer agreement with Real). For the GAN and diffusion models, blending consistently reduced the JSD relative to No-Blend across all species, splice categories, and both sequence lengths, confirming that frequency blending brings the k-mer composition of these sequences closer to that of real sequences. The effect was largest for the GAN model (for example, *A. thaliana* donor at k=6: from 0.2994 to 0.2296). For the VAE model, the JSD was already low without blending and changed only marginally with blending, consistent with its strong unblended predictive performance in the other evaluations. Across all conditions, blended diffusion-generated sequences achieved the lowest JSD, indicating the closest k-mer composition to real sequences among the 3 models. These quantitative results corroborate the descriptive analyses and provide a statistical measure of distributional similarity.

**Table 6.** Quantitative comparison of *k*-mer spectra between real and synthetic sequences, measured by Jensen–Shannon divergence (JSD; lower indicates closer agreement with Real) for k=3 and k=6. Values are reported for the GAN, VAE, and diffusion models without blending (No-Blend, λ=0) and with blending (Blend, λ=0.5), for A*rabidopsis thaliana*, *Homo sapiens*, and *Danio rerio* at both sequence lengths.

Species	Site	Length	k=3						k=6					
			No-Blend			Blend			No-Blend			Blend		
			GAN	VAE	Diff	GAN	VAE	Diff	GAN	VAE	Diff	GAN	VAE	Diff
*Arabidopsis*	Donor	402	0.1951	0.1107	0.0544	0.1376	0.1159	0.0386	0.2994	0.1984	0.1254	0.2296	0.2018	0.1167
		2,002	0.1928	0.1008	0.0450	0.1305	0.1115	0.0320	0.2900	0.1775	0.1005	0.2129	0.1885	0.0886
	Acceptor	402	0.1866	0.1059	0.0537	0.1317	0.1080	0.0409	0.2882	0.1901	0.1229	0.2215	0.1919	0.1143
		2,002	0.1916	0.1013	0.0464	0.1305	0.1106	0.0343	0.2885	0.1771	0.1010	0.2124	0.1871	0.0898
*Homo sapiens*	Donor	402	0.1626	0.1653	0.0602	0.1609	0.1621	0.0563	0.2872	0.2890	0.1318	0.2852	0.2849	0.1318
		2,002	0.1681	0.1666	0.0649	0.1642	0.1733	0.0601	0.2904	0.2892	0.1278	0.2857	0.2956	0.1234
	Acceptor	402	0.1740	0.1593	0.0584	0.1625	0.1611	0.0486	0.2978	0.2775	0.1304	0.2837	0.2794	0.1214
		2,002	0.1815	0.1673	0.0588	0.1693	0.1708	0.0456	0.3061	0.2876	0.1257	0.2915	0.2916	0.1118
*Danio rerio*	Donor	402	0.1798	0.1272	0.0679	0.1404	0.1319	0.0541	0.2967	0.2394	0.1513	0.2535	0.2425	0.1447
		2,002	0.2050	0.1151	0.0558	0.1443	0.1225	0.0405	0.3139	0.2152	0.1246	0.2451	0.2222	0.1115
	Acceptor	402	0.1799	0.1292	0.0672	0.1466	0.1343	0.0415	0.2971	0.2370	0.1494	0.2592	0.2442	0.1280
		2,002	0.2072	0.1169	0.0576	0.1456	0.1224	0.0351	0.3167	0.2153	0.1240	0.2466	0.2211	0.1045

GAN, generative adversarial network; VAE, variational autoencoder; Diff, diffusion

To quantify the PPT deficiency noted in the discussion, we scored acceptor sequences over positions −40 to −5 relative to AG, measuring tract severity as the mean pyrimidine (C/T) fraction, tract integrity as the mean longest consecutive pyrimidine run, and malformation frequency as the fraction of sequences with pyrimidine content below the 5th percentile of Real (Table 7). Without blending, GAN- and diffusion-generated sequences produced pyrimidine-poor tracts; for *H. sapiens*, the diffusion model reached a mean pyrimidine fraction of only 0.495 (versus 0.633 for Real), with a malformation rate of 26.7%. Blending substantially mitigated this deficiency, raising the fraction to 0.651 and reducing the malformation rate to 1.1%, with comparable improvements for GAN and across the other species; VAE-generated sequences remained close to Real regardless of blending (malformation rates below 1%). The integrity measure showed the same mitigation for GAN, which stayed below Real, but revealed that blended diffusion-generated sequences produced pyrimidine runs longer than those of real sequences (*H. sapiens*: 10.81 versus 7.98), consistent with the oversharpening of upstream pyrimidine content seen in the conservation and sequence-logo analyses. Overall, frequency blending partially mitigates PPT malformation while, for the diffusion model, overshooting tract integrity, indicating that the deficiency is reduced but not perfectly corrected.

**Table 7.** Polypyrimidine tract (PPT) pyrimidine fraction (severity) and longest consecutive pyrimidine run (integrity) at acceptor sites, scored over positions −40 to −5 relative to AG, for Real, No-Blend (λ=0), and Blend (λ=0.5) sequences. Values closer to Real indicate better-formed tracts; malformation rates are reported in the text. Values were equivalent at 402 and 2,002 bp.

Species	Real	No-Blend			Blend		
		GAN	VAE	Diff	GAN	VAE	Diff
Pyrimidine fraction							
*Arabidopsis*	0.586	0.520	0.614	0.503	0.565	0.617	0.620
*Homo sapiens*	0.633	0.516	0.638	0.495	0.579	0.649	0.651
*Danio rerio*	0.612	0.524	0.617	0.509	0.569	0.603	0.665
Longest pyrimidine run							
*Arabidopsis*	6.10	4.79	6.34	4.97	5.43	6.34	7.68
*Homo sapiens*	7.98	4.75	7.32	5.03	5.72	7.35	10.81
*Danio rerio*	6.72	4.81	6.69	4.84	5.53	6.25	8.95

GAN, generative adversarial network; VAE, variational autoencoder; Diff, diffusion

### Indirect evaluation

We divided the indirect evaluation into 3 complementary scenarios, each designed to assess a distinct aspect of the quality and utility of the synthetic sequences. For each scenario, we used models trained and tested on real sequences (Train-Real/Test-Real) as the baseline for comparison.

#### Scenario 1: Train-Real/Test-Synthetic

Table 8 shows evaluation results for *A. thaliana* and *H. sapiens*. In this scenario, we trained splice site classifiers (SpliceRover and Spliceator) exclusively on real sequences and tested them on synthetic sequences. This protocol quantifies the biological realism and fidelity of synthetic sequences by evaluating whether they preserve the discriminative patterns that classifiers learn from real sequences.

**Table 8.** Scenario 1 (Train-Real/Test-Synthetic). Predictive performance of SpliceRover and Spliceator proxy models on synthetic sequences generated by GAN, VAE, and diffusion models for *Arabidopsis thaliana*, *Homo sapiens*, and *Danio rerio*. Baseline values (Train-Real/Test-Real) are shown in parentheses.

Species	Metric (baseline)	No-Blend			Blend		
		GAN	VAE	Diff	GAN	VAE	Diff
Donor—SpliceRover							
*Arabidopsis*	F1 (0.9595)	0.0538	0.8395	0.1656	0.6075	0.8858	0.8358
	MCC (0.9188)	−0.0401	0.7261	0.0993	0.4754	0.7923	0.7211
	AUROC (0.9899)	0.6036	0.9365	0.5992	0.8416	0.9578	0.9512
*Homo sapiens*	F1 (0.9600)	0.1808	0.9118	0.2272	0.7414	0.9294	0.9638
	MCC (0.9191)	0.0917	0.8305	0.1365	0.5985	0.8612	0.9269
	AUROC (0.9916)	0.7668	0.9750	0.6834	0.9165	0.9817	0.9925
*Danio rerio*	F1 (0.9837)	0.1901	0.8771	0.1300	0.5894	0.9110	0.9125
	MCC (0.9674)	0.1500	0.7846	0.0901	0.4734	0.8359	0.8384
	AUROC (0.9983)	0.6336	0.9620	0.5366	0.8475	0.9738	0.9785
Donor—Spliceator							
*Arabidopsis*	F1 (0.9598)	0.1213	0.8672	0.2100	0.6764	0.9062	0.8864
	MCC (0.9191)	0.0435	0.7631	0.1353	0.5369	0.8233	0.7920
	AUROC (0.9904)	0.7408	0.9499	0.6848	0.8838	0.9701	0.9662
*Homo sapiens*	F1 (0.9577)	0.3212	0.9099	0.2164	0.7279	0.9255	0.9644
	MCC (0.9146)	0.2127	0.8262	0.1190	0.5804	0.8535	0.9282
	AUROC (0.9900)	0.8166	0.9735	0.6534	0.9071	0.9798	0.9921
*Danio rerio*	F1 (0.9812)	0.2099	0.9047	0.1482	0.6378	0.9304	0.9397
	MCC (0.9624)	0.1561	0.8242	0.0964	0.5102	0.8666	0.8828
	AUROC (0.9975)	0.6945	0.9736	0.5964	0.8857	0.9820	0.9848
Acceptor—SpliceRover							
*Arabidopsis*	F1 (0.9573)	0.1206	0.8687	0.2501	0.6328	0.8958	0.8376
	MCC (0.9140)	0.0291	0.7626	0.1599	0.4884	0.8045	0.7182
	AUROC (0.9904)	0.6829	0.9495	0.6488	0.8604	0.9641	0.9370
*Homo sapiens*	F1 (0.9591)	0.2143	0.8391	0.1296	0.5842	0.8621	0.8201
	MCC (0.9192)	0.1749	0.7332	0.0933	0.4706	0.7640	0.7089
	AUROC (0.9890)	0.7979	0.9628	0.6548	0.8955	0.9679	0.9527
*Danio rerio*	F1 (0.9831)	0.1115	0.8164	0.0924	0.4536	0.7974	0.9288
	MCC (0.9661)	0.0740	0.7046	0.0508	0.3656	0.6817	0.8656
	AUROC (0.9977)	0.5899	0.9531	0.5262	0.8152	0.9448	0.9869
Acceptor—Spliceator							
*Arabidopsis*	F1 (0.9565)	0.0971	0.8716	0.2320	0.5757	0.8973	0.8434
	MCC (0.9126)	0.0067	0.7682	0.1493	0.4394	0.8080	0.7278
	AUROC (0.9883)	0.6794	0.9564	0.6437	0.8557	0.9677	0.9418
*Homo sapiens*	F1 (0.9532)	0.2713	0.8435	0.1067	0.5560	0.8608	0.8446
	MCC (0.9088)	0.2303	0.7410	0.0773	0.4517	0.7642	0.7425
	AUROC (0.9882)	0.8300	0.9694	0.6469	0.9000	0.9738	0.9642
*Danio rerio*	F1 (0.9778)	0.1297	0.8937	0.1073	0.5454	0.8778	0.9425
	MCC (0.9554)	0.0777	0.8071	0.0518	0.4301	0.7834	0.8879
	AUROC (0.9958)	0.6569	0.9745	0.5494	0.8749	0.9706	0.9874

GAN, generative adversarial network; VAE, variational autoencoder; Diff, diffusion; MCC, Matthews correlation coefficient; AUROC, area under the receiver operating characteristic curve

Blending produces the most substantial improvements for sequences generated by the diffusion and GAN models. For *A. thaliana* donor sites, when tested on diffusion-generated sequences, SpliceRover shows large improvements in predictive performance with blending: F1-score increases from 0.1656 (No-Blend) to 0.8358 (Blend), MCC from 0.0993 to 0.7211, and AUROC from 0.5992 to 0.9512 (baseline: F1-score 0.9595, MCC 0.9188, and AUROC 0.9899). GAN-generated sequences show similar substantial gains: F1-score from 0.0538 to 0.6075, MCC from −0.0401 to 0.4754, and AUROC from 0.6036 to 0.8416. Spliceator demonstrates comparable improvements. In contrast, VAE-generated sequences achieve high predictive performance even without blending (e.g., Spliceator: F1-score 0.8672, MCC 0.7631, and AUROC 0.9499 for No-Blend).

For *H. sapiens* donor sites, diffusion-generated sequences with blending achieve near-baseline performance: F1-score 0.9638, MCC 0.9269, and AUROC 0.9925 (baseline: F1-score 0.9600, MCC 0.9191, and AUROC 0.9916), compared to F1-score 0.2272, MCC 0.1365, and AUROC 0.6834 without blending. The same pattern holds for *D. rerio* donor sites: diffusion-generated sequences improve markedly with blending, with SpliceRover F1-score increasing from 0.1300 (No-Blend) to 0.9125 (Blend), MCC from 0.0901 to 0.8384, and AUROC from 0.5366 to 0.9785 (baseline: F1-score 0.9837, MCC 0.9674, and AUROC 0.9983), while GAN-generated sequences also show substantial gains (F1-score from 0.1901 to 0.5894, MCC from 0.1500 to 0.4734, and AUROC from 0.6336 to 0.8475), and VAE-generated sequences again perform well irrespective of blending (F1-score 0.8771 and AUROC 0.9620 for No-Blend). Acceptor sites show similar patterns, with diffusion and GAN models benefiting substantially from blending, while VAE-generated sequences maintain consistently high predictive performance. For *D. rerio* acceptor sites in particular, blended diffusion-generated sequences reach the highest performance among all generators (SpliceRover F1-score 0.9288 and AUROC 0.9869), exceeding the corresponding VAE values. Overall, frequency blending substantially improves biological fidelity for sequences generated by the diffusion and GAN models, whereas VAE-generated sequences maintain high predictive performance with or without blending.

Table 9 shows the Train-Real/Test-Synthetic results for the extended-context 2,002-bp sequences, using Spliceator as the proxy classifier. The patterns observed at 402 bp are preserved at this longer context across all 3 species. Sequences generated by the GAN and diffusion models are poorly recognized without blending but improve substantially with it; for example, on *H. sapiens* donor sites, the predictive performance of the diffusion model rises from an F1-score of 0.0901 (No-Blend) to 0.9531 (Blend) and an AUROC of 0.5543 to 0.9931 (baseline F1-score 0.9371 and AUROC 0.9860). VAE-generated sequences again perform well irrespective of blending. Notably, blended diffusion-generated sequences reach the highest predictive performance among all generators on several species and splice categories, matching or exceeding the corresponding VAE values. The consistency of these results with the 402-bp setting indicates that the benefits of frequency blending for the GAN and diffusion models, and its limited effect on the VAE, are not specific to a particular sequence length.

**Table 9.** Scenario 1 (Train-Real/Test-Synthetic) for the extended-context 2,002-bp sequences. Predictive performance of the Spliceator proxy model on synthetic sequences generated by the GAN, VAE, and diffusion models for *Arabidopsis thaliana*, *Homo sapiens*, and *Danio rerio*. SpliceRover is not included because its architecture is defined only for sequences up to 400 bp. Baseline values (Train-Real/Test-Real) are shown in parentheses.

Species	Metric (baseline)	No-Blend			Blend		
		GAN	VAE	Diff	GAN	VAE	Diff
Donor—Spliceator							
*Arabidopsis*	F1 (0.9378)	0.1260	0.9121	0.1221	0.6136	0.9243	0.9476
	MCC (0.8801)	0.0742	0.8362	0.0697	0.4892	0.8565	0.8971
	AUROC (0.9743)	0.5895	0.9702	0.5595	0.8672	0.9749	0.9814
*Homo sapiens*	F1 (0.9371)	0.2654	0.9003	0.0901	0.6352	0.9111	0.9531
	MCC (0.8804)	0.2205	0.8201	0.0501	0.5166	0.8370	0.9083
	AUROC (0.9860)	0.7509	0.9772	0.5543	0.8963	0.9804	0.9931
*Danio rerio*	F1 (0.9603)	0.1593	0.8733	0.0779	0.5597	0.9051	0.9275
	MCC (0.9225)	0.1514	0.7849	0.0626	0.4638	0.8311	0.8662
	AUROC (0.9858)	0.7618	0.9730	0.5634	0.8889	0.9810	0.9880
Acceptor—Spliceator							
*Arabidopsis*	F1 (0.8990)	0.0639	0.8892	0.0969	0.4887	0.8913	0.9430
	MCC (0.8180)	0.0118	0.8031	0.0569	0.3934	0.8062	0.8901
	AUROC (0.9674)	0.5712	0.9723	0.5697	0.8499	0.9737	0.9855
*Homo sapiens*	F1 (0.9089)	0.2296	0.9024	0.1230	0.6220	0.9186	0.9548
	MCC (0.8286)	0.1578	0.8179	0.0505	0.4873	0.8447	0.9097
	AUROC (0.9725)	0.6955	0.9718	0.5432	0.8733	0.9778	0.9886
*Danio rerio*	F1 (0.9404)	0.1384	0.9000	0.0912	0.5698	0.8846	0.9471
	MCC (0.8839)	0.0931	0.8179	0.0378	0.4537	0.7944	0.8967
	AUROC (0.9769)	0.6298	0.9750	0.5083	0.8718	0.9711	0.9898

GAN, generative adversarial network; VAE, variational autoencoder; Diff, diffusion; MCC, Matthews correlation coefficient; AUROC, area under the receiver operating characteristic curve

**Fig. 5.**
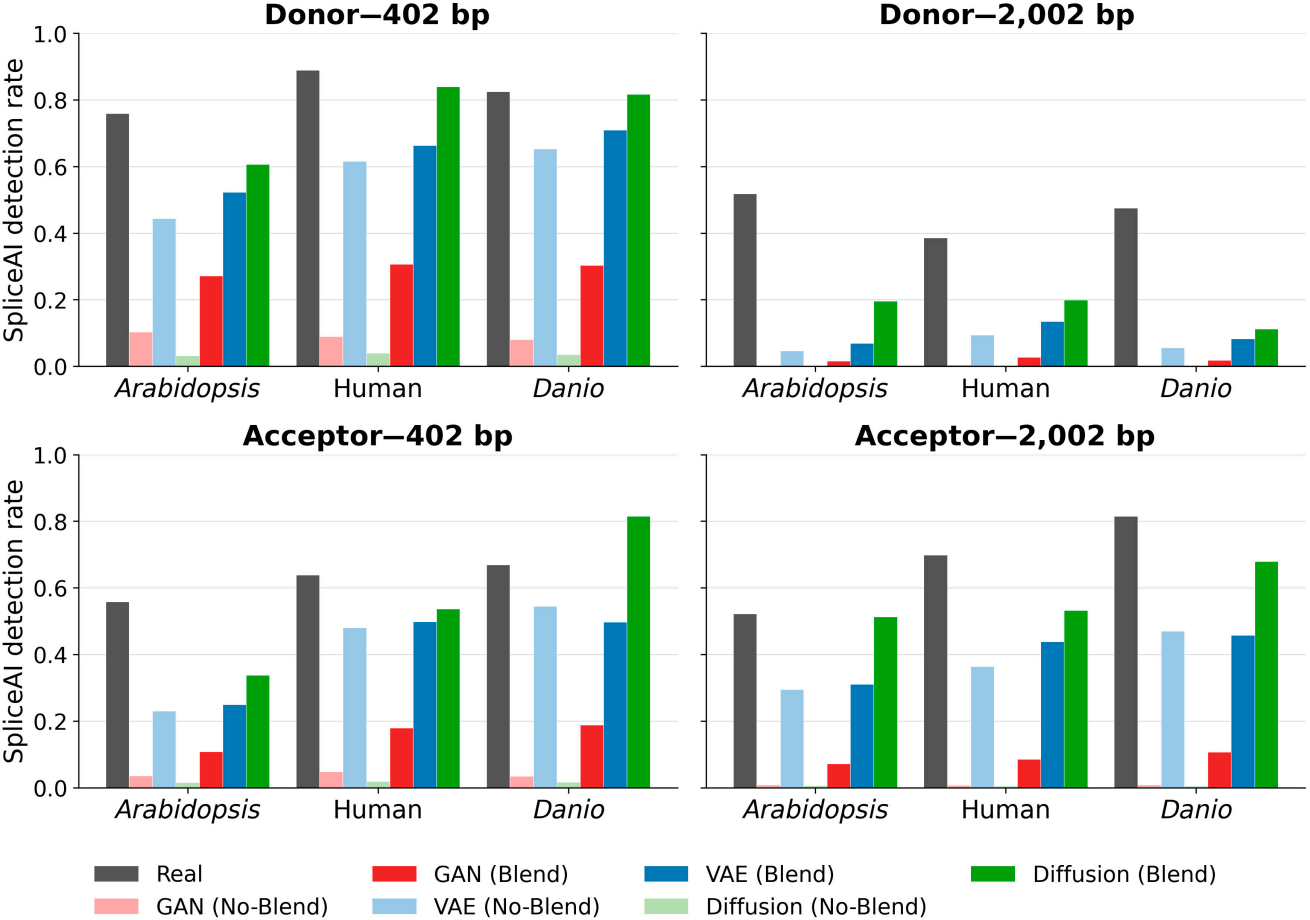
SpliceAI detection rate at a fixed junction position (τ=0.5), used as an independent external validator, for the 402-bp and extended-context 2,002-bp sequences. Each panel shows the 3 species; bars give the detection rate for real sequences (Real) and for synthetic sequences generated by the generative adversarial network (GAN), variational autoencoder (VAE), and diffusion models, with and without blending (λ=0 and λ=0.5). Detection rates should be interpreted relative to the real-sequence reference, which differs between sequence-length settings.

Figure 5 shows the SpliceAI detection rate at the fixed junction position for both sequence lengths. As an independent validator that shares neither architecture nor training data with the proxy classifiers, SpliceAI reproduces the same qualitative pattern observed earlier: blending markedly increases the detection of GAN- and diffusion-generated sequences, whereas VAE-generated sequences are already detected at higher rates without blending. Among the generators, blended diffusion-generated sequences consistently achieve the highest detection, approaching the real-sequence reference. Detection rates are interpreted relative to this reference, which itself differs between the 2 settings: SpliceAI detects real 2,002-bp sequences at lower rates than real 402-bp sequences, so the lower absolute detection of synthetic 2,002-bp sequences reflects this shifted reference rather than a failure of generation. The agreement between SpliceAI and the proxy classifiers, despite their full independence, indicates that the high proxy predictive performance reflects genuine biological realism rather than feature overlap specific to SpliceRover and Spliceator.

#### Scenario 2: Train-Synthetic/Test-Real

Table 10 presents the results of scenario 2 for *A. thaliana* and *H. sapiens*. In this scenario, we trained splice site classifiers (SpliceRover and Spliceator) exclusively on synthetic sequences and tested them on real sequences. This protocol quantifies the domain shift between synthetic- and real-sequence distributions and establishes whether frequency blending enhances model generalization and transferability to real genomic sequences.

**Table 10.** Scenario 2(Train-Synthetic/Test-Real). Predictive performance of SpliceRover and Spliceator trained on synthetic sequences and evaluated on real sequences for *Arabidopsis thaliana*, *Homo sapiens*, and *Danio rerio*. Baseline values (Train-Real/Test-Real) shown in parentheses.

Species	Metric (baseline)	No-Blend			Blend		
		GAN	VAE	Diff	GAN	VAE	Diff
Donor—SpliceRover							
*Arabidopsis*	F1 (0.9595)	0.0329	0.4023	0.3390	0.2296	0.4221	0.6906
	MCC (0.9188)	−0.0266	0.3381	0.1703	0.1948	0.3661	0.5691
	AUROC (0.9899)	0.6437	0.8367	0.6967	0.7602	0.8646	0.8849
*Homo sapiens*	F1 (0.9600)	0.0234	0.0188	0.1334	0.0411	0.1157	0.2528
	MCC (0.9191)	0.0435	0.0402	0.0846	0.0580	0.1251	0.2717
	AUROC (0.9916)	0.6092	0.5842	0.5190	0.5855	0.6514	0.8018
*Danio rerio*	F1 (0.9837)	0.0539	0.7014	0.2016	0.1344	0.6497	0.7255
	MCC (0.9674)	−0.0238	0.5293	0.0864	0.0688	0.4804	0.6141
	AUROC (0.9983)	0.5478	0.8483	0.6460	0.6711	0.8452	0.9033
Donor—Spliceator							
*Arabidopsis*	F1 (0.9598)	0.0409	0.3322	0.3381	0.2677	0.3526	0.6795
	MCC (0.9191)	0.0085	0.3106	0.1877	0.2200	0.3273	0.5615
	AUROC (0.9904)	0.6530	0.8554	0.7205	0.7905	0.8686	0.9005
*Homo sapiens*	F1 (0.9577)	0.0193	0.0653	0.1528	0.0190	0.0765	0.2181
	MCC (0.9146)	0.0454	0.1109	0.0753	0.0418	0.1279	0.2474
	AUROC (0.9900)	0.4874	0.7454	0.5874	0.5817	0.7535	0.7065
*Danio rerio*	F1 (0.9812)	0.1111	0.7345	0.3857	0.3728	0.8036	0.8096
	MCC (0.9624)	−0.0029	0.5670	0.1568	0.1850	0.6234	0.6958
	AUROC (0.9975)	0.5794	0.8728	0.6703	0.6893	0.8906	0.9394
Acceptor—SpliceRover							
*Arabidopsis*	F1 (0.9573)	0.0081	0.4472	0.4089	0.2093	0.4977	0.6170
	MCC (0.9140)	−0.0545	0.3761	0.2422	0.1865	0.4199	0.5170
	AUROC (0.9904)	0.6421	0.8563	0.7472	0.7850	0.8944	0.9062
*Homo sapiens*	F1 (0.9591)	0.0148	0.0176	0.1241	0.0092	0.0406	0.2677
	MCC (0.9192)	0.0231	0.0479	0.0649	0.0283	0.0909	0.2673
	AUROC (0.9890)	0.5370	0.6641	0.5534	0.5044	0.6558	0.7354
*Danio rerio*	F1 (0.9831)	0.2675	0.8347	0.3454	0.1958	0.7287	0.6641
	MCC (0.9661)	−0.0478	0.7200	0.1137	0.1505	0.5769	0.5566
	AUROC (0.9977)	0.4971	0.9374	0.6276	0.7026	0.8767	0.8955
Acceptor—Spliceator							
*Arabidopsis*	F1 (0.9565)	0.0147	0.7112	0.4597	0.1979	0.6651	0.7066
	MCC (0.9126)	−0.0409	0.5709	0.2690	0.1766	0.5405	0.5902
	AUROC (0.9883)	0.6542	0.8998	0.7490	0.7825	0.9076	0.9271
*Homo sapiens*	F1 (0.9532)	0.0092	0.0410	0.1040	0.0102	0.0567	0.2282
	MCC (0.9088)	0.0216	0.0875	0.0358	0.0373	0.1084	0.2468
	AUROC (0.9882)	0.5084	0.6888	0.5011	0.6490	0.7043	0.7679
*Danio rerio*	F1 (0.9778)	0.0340	0.8280	0.4293	0.2918	0.6778	0.6947
	MCC (0.9554)	−0.0493	0.6947	0.1431	0.2026	0.5606	0.5898
	AUROC (0.9958)	0.5977	0.9271	0.6283	0.7431	0.9020	0.9435

GAN, generative adversarial network; VAE, variational autoencoder; Diff, diffusion; MCC, Matthews correlation coefficient; AUROC, area under the receiver operating characteristic curve

For *A. thaliana*, blending produces substantial improvements, particularly for diffusion-generated sequences. For donor sites, when trained on diffusion-generated sequences with blending, SpliceRover achieves F1-score 0.6906, MCC 0.5691, and AUROC 0.8849 (baseline: F1-score 0.9595, MCC 0.9188, and AUROC 0.9899), compared to F1-score 0.3390, MCC 0.1703, and AUROC 0.6967 without blending. For acceptor sites, SpliceRover trained on diffusion-generated sequences with blending reaches F1-score 0.6170, MCC 0.5170, and AUROC 0.9062(baseline: F1-score 0.9573, MCC 0.9140, and AUROC 0.9904). VAE-generated sequences achieve moderate predictive performance with blending, while GAN-generated sequences show the weakest transferability. Spliceator demonstrates similar trends.

For *H. sapiens*, synthetic-to-real transfer proves substantially more challenging, with large gaps in predictive performance remaining even with blending. For donor sites, SpliceRover trained on diffusion-generated sequences with blending achieves F1-score 0.2528, MCC 0.2717, and AUROC 0.8018 (baseline: F1-score 0.9600, MCC 0.9191, and AUROC 0.9916), compared to F1-score 0.1334, MCC 0.0846, and AUROC 0.5190 without blending. Similar patterns emerge for acceptor sites across both classifiers.

For *D. rerio*, synthetic-to-real transfer is comparatively strong, exceeding that of *H. sapiens* and approaching the levels observed for *A. thaliana*. For donor sites, Spliceator trained on diffusion-generated sequences with blending achieves F1-score 0.8096, MCC 0.6958, and AUROC 0.9394 (baseline: F1-score 0.9812, MCC 0.9624, and AUROC 0.9975), compared to F1-score 0.3857, MCC 0.1568, and AUROC 0.6703 without blending. For acceptor sites, Spliceator trained on diffusion-generated sequences with blending reaches F1-score 0.6947, MCC 0.5898, and AUROC 0.9435. As for the other species, VAE-generated sequences transfer well with little dependence on blending, while GAN-generated sequences show the weakest transferability.

Overall, frequency blending improves synthetic-to-real transfer, with diffusion-generated sequences consistently achieving the strongest results. However, the persistent gap in predictive performance indicates that synthetic sequences do not fully replicate the complexity of real genomic sequences, highlighting fundamental limits in current generative modeling approaches for biological data.

Table 11 shows the Train-Synthetic/Test-Real results for the extended-context 2,002-bp sequences, using Spliceator as the proxy classifier. The trends observed at 402 bp are preserved: frequency blending improves synthetic-to-real transfer, with diffusion-generated sequences consistently achieving the strongest results, while GAN- and VAE-generated sequences transfer less effectively. However, even with blending, a substantial gap in predictive performance relative to the real-sequence baseline remains, and transfer to real *H. sapiens* sequences continues to be the most challenging. As at the shorter context, these results indicate that synthetic sequences do not fully replicate the complexity of real genomic sequences, and that this limitation persists and is in some cases more pronounced, at the longer context.

**Table 11.** Scenario 2 (Train-Synthetic/Test-Real) for the extended-context 2,002-bp sequences. Predictive performance of Spliceator trained on synthetic sequences and evaluated on real sequences for *Arabidopsis thaliana*, *Homo sapiens*, and *Danio rerio*. Baseline values (Train-Real/Test-Real) are shown in parentheses. SpliceRover is not included because its architecture is defined only for sequences up to 400 bp.

Species	Metric (baseline)	No-Blend			Blend		
		GAN	VAE	Diff	GAN	VAE	Diff
Donor—Spliceator							
*Arabidopsis*	F1 (0.9378)	0.0046	0.0915	0.3211	0.0144	0.0598	0.7399
	MCC (0.8801)	−0.0144	0.1062	0.0439	−0.0110	0.0635	0.6240
	AUROC (0.9743)	0.4916	0.6876	0.5220	0.5225	0.6223	0.9324
*Homo sapiens*	F1 (0.9371)	0.0485	0.0530	0.0743	0.0044	0.0008	0.1658
	MCC (0.8804)	0.1038	0.0972	0.0607	0.0197	−0.0098	0.1699
	AUROC (0.9860)	0.6272	0.7038	0.6970	0.6466	0.6662	0.7342
*Danio rerio*	F1 (0.9603)	0.0468	0.0247	0.3089	0.0793	0.0925	0.7570
	MCC (0.9225)	−0.0171	0.0326	0.0294	0.0040	0.1210	0.6428
	AUROC (0.9858)	0.5484	0.6461	0.5473	0.5539	0.6989	0.9270
Acceptor—Spliceator							
*Arabidopsis*	F1 (0.8990)	0.0018	0.0644	0.1760	0.1168	0.2588	0.7070
	MCC (0.8180)	−0.0212	0.1101	0.0000	0.0589	0.2394	0.5897
	AUROC (0.9674)	0.4836	0.7560	0.4904	0.6029	0.7702	0.9401
*Homo sapiens*	F1 (0.9089)	0.0000	0.2002	0.2036	0.0589	0.0436	0.7163
	MCC (0.8286)	0.0000	0.1590	−0.0116	0.0689	0.0765	0.6017
	AUROC (0.9725)	0.5000	0.6176	0.5157	0.6360	0.6783	0.9373
*Danio rerio*	F1 (0.9404)	0.0176	0.3472	0.0000	0.0627	0.3812	0.5651
	MCC (0.8839)	−0.0581	0.2983	0.0000	0.0289	0.3032	0.4766
	AUROC (0.9769)	0.5000	0.7783	0.5000	0.5447	0.7519	0.8487

GAN, generative adversarial network; VAE, variational autoencoder; Diff, diffusion; MCC, Matthews correlation coefficient; AUROC, area under the receiver operating characteristic curve

#### Scenario 3: Data augmentation

Table 12 presents the results of scenario 3 for *A. thaliana* and *H. sapiens*. In this scenario, we trained splice site classifiers on fixed-size datasets where real sequences (at 10%, 25%, and 50% of the training set) were augmented with synthetic sequences (generated with λ=0.5). Models were evaluated on real test sequences to assess whether augmenting limited real sequences with synthetic sequences improves predictive performance. The baseline (100% real sequences) represents the upper bound of achievable predictive performance.

**Table 12.** Scenario 3 (data augmentation). Models trained on mixtures of real and synthetic sequences and tested on real sequences for *Arabidopsis thaliana*, *Homo sapiens*, and *Danio rerio*. Baseline (100% real) shown in parentheses.

Species	Metric (baseline)	GAN			VAE			Diffusion		
		10%	25%	50%	10%	25%	50%	10%	25%	50%
Donor—SpliceRover										
*Arabidopsis*	F1 (0.9595)	0.9246	0.9518	0.9577	0.9274	0.9491	0.9577	0.9008	0.9408	0.9516
	MCC (0.9188)	0.8590	0.9048	0.9164	0.8674	0.8998	0.9168	0.8237	0.8846	0.9028
	AUROC (0.9899)	0.9779	0.9839	0.9872	0.9836	0.9842	0.9875	0.9766	0.9830	0.9866
*Homo sapiens*	F1 (0.9600)	0.8939	0.9329	0.9603	0.9139	0.9562	0.9572	0.9405	0.9474	0.9500
	MCC (0.9191)	0.8187	0.8760	0.9202	0.8464	0.9138	0.9162	0.8866	0.8994	0.9034
	AUROC (0.9916)	0.9863	0.9879	0.9899	0.9861	0.9899	0.9900	0.9846	0.9890	0.9902
*Danio rerio*	F1 (0.9837)	0.9424	0.9511	0.9585	0.9478	0.9575	0.9594	0.9465	0.9570	0.9614
	MCC (0.9674)	0.8871	0.9053	0.9179	0.8972	0.9161	0.9198	0.8973	0.9163	0.9239
	AUROC (0.9983)	0.9773	0.9826	0.9842	0.9780	0.9833	0.9839	0.9797	0.9825	0.9845
Donor—Spliceator										
*Arabidopsis*	F1 (0.9598)	0.9122	0.9448	0.9429	0.9153	0.9423	0.9462	0.9262	0.9397	0.9506
	MCC (0.9191)	0.8309	0.8906	0.8851	0.8430	0.8877	0.8928	0.8587	0.8813	0.9014
	AUROC (0.9904)	0.9676	0.9828	0.9824	0.9764	0.9828	0.9844	0.9772	0.9817	0.9865
*Homo sapiens*	F1 (0.9577)	0.9124	0.9412	0.9448	0.8838	0.9282	0.9463	0.9006	0.9448	0.9508
	MCC (0.9146)	0.8420	0.8872	0.8926	0.8038	0.8675	0.8966	0.8259	0.8932	0.9036
	AUROC (0.9900)	0.9851	0.9861	0.9872	0.9860	0.9881	0.9889	0.9853	0.9879	0.9880
*Danio rerio*	F1 (0.9812)	0.9226	0.9496	0.9544	0.9267	0.9516	0.9554	0.9297	0.9540	0.9569
	MCC (0.9624)	0.8485	0.9004	0.9086	0.8605	0.9041	0.9109	0.8668	0.9098	0.9139
	AUROC (0.9975)	0.9703	0.9823	0.9837	0.9767	0.9811	0.9833	0.9769	0.9812	0.9839
Acceptor—SpliceRover										
*Arabidopsis*	F1 (0.9573)	0.8957	0.9290	0.9422	0.8767	0.9386	0.9451	0.9064	0.9357	0.9518
	MCC (0.9140)	0.8127	0.8656	0.8850	0.7914	0.8834	0.8944	0.8319	0.8796	0.9040
	AUROC (0.9904)	0.9719	0.9796	0.9810	0.9769	0.9841	0.9848	0.9764	0.9850	0.9859
*Homo sapiens*	F1 (0.9591)	0.8958	0.8677	0.9502	0.8332	0.9159	0.9443	0.8536	0.9235	0.9524
	MCC (0.9192)	0.8216	0.7835	0.9040	0.7408	0.8506	0.8944	0.7629	0.8586	0.9067
	AUROC (0.9890)	0.9838	0.9848	0.9870	0.9835	0.9864	0.9877	0.9802	0.9822	0.9870
*Danio rerio*	F1 (0.9831)	0.9185	0.9397	0.9513	0.9326	0.9452	0.9492	0.9347	0.9454	0.9506
	MCC (0.9661)	0.8454	0.8846	0.9032	0.8719	0.8925	0.9003	0.8759	0.8930	0.9016
	AUROC (0.9977)	0.9689	0.9793	0.9828	0.9760	0.9799	0.9815	0.9739	0.9799	0.9840
Acceptor—Spliceator										
*Arabidopsis*	F1 (0.9565)	0.8860	0.9273	0.9364	0.9073	0.9310	0.9439	0.8811	0.9270	0.9454
	MCC (0.9126)	0.7880	0.8595	0.8726	0.8304	0.8679	0.8899	0.7946	0.8599	0.8924
	AUROC (0.9883)	0.9590	0.9771	0.9793	0.9728	0.9795	0.9845	0.9737	0.9791	0.9840
*Homo sapiens*	F1 (0.9532)	0.8269	0.9083	0.9408	0.8061	0.9024	0.9379	0.8493	0.8898	0.9457
	MCC (0.9088)	0.7326	0.8390	0.8879	0.7088	0.8304	0.8835	0.7547	0.8104	0.8944
	AUROC (0.9882)	0.9800	0.9839	0.9861	0.9796	0.9848	0.9853	0.9728	0.9808	0.9845
*Danio rerio*	F1 (0.9778)	0.9124	0.9350	0.9454	0.9218	0.9376	0.9430	0.9281	0.9433	0.9502
	MCC (0.9554)	0.8369	0.8716	0.8922	0.8538	0.8798	0.8858	0.8645	0.8881	0.9024
	AUROC (0.9958)	0.9709	0.9758	0.9800	0.9742	0.9791	0.9781	0.9749	0.9793	0.9824

GAN, generative adversarial network; VAE, variational autoencoder; MCC, Matthews correlation coefficient; AUROC, area under the receiver operating characteristic curve

Augmenting 50% real sequences with 50% synthetic sequences consistently achieves near-baseline performance across all 3 species, classifiers, and generative architectures. For *A. thaliana* donor sites (baseline for SpliceRover: F1-score 0.9595, MCC 0.9188, and AUROC 0.9899), all 3 generators at 50% achieve strong results with SpliceRover: GAN-augmented training reaches F1-score 0.9577, MCC 0.9164, and AUROC 0.9872; VAE-augmented training achieves F1-score 0.9577, MCC 0.9168, and AUROC 0.9875; diffusion-augmented training attains F1-score 0.9516, MCC 0.9028, and AUROC 0.9866. Comparable results are observed with Spliceator. For *H. sapiens* donor sites (baseline for SpliceRover: F1-score 0.9600, MCC 0.9191, and AUROC 0.9916), augmentation with GAN-generated sequences exceeds baseline with SpliceRover: F1-score 0.9603, MCC 0.9202, and AUROC 0.9899. Similar patterns emerge across acceptor sites and both classifier architectures. For *D. rerio* donor sites (baseline for SpliceRover: F1-score 0.9837, MCC 0.9674, and AUROC 0.9983), the 50% mixtures likewise reach near-baseline performance: diffusion-augmented training attains F1-score 0.9614, MCC 0.9239, and AUROC 0.9845, with GAN- and VAE-augmented training giving comparable values, and Spliceator shows the same trend.

Lower real-sequence fractions show progressive degradation in predictive performance. At 25% real augmented with 75% synthetic, classifiers retain reasonable predictive performance with noticeable gaps. At 10% real augmented with 90% synthetic, predictive performance degrades substantially, particularly for *H. sapiens* acceptor sites where SpliceRover F1-scores range from 0.8332 (VAE) to 0.8958 (GAN) compared to baseline 0.9591. The same degradation at 10% real is also observed for *D. rerio*, although it remains less pronounced than for *H. sapiens* (for example, SpliceRover acceptor F1-score 0.9185 for GAN-augmented training compared to baseline 0.9831). Overall, augmenting 50% real training sequences with 50% synthetic sequences provides an effective strategy for achieving near-baseline predictive performance while halving real-sequence requirements.

Table 13 shows the data augmentation results for the extended-context 2,002-bp sequences, using Spliceator trained on mixtures of real and synthetic sequences. As at 402 bp, augmenting 50% real sequences with 50% synthetic sequences achieves predictive performance close to that of the 100% real baseline across all 3 species, both splice categories, and all 3 generators; for example, on *H. sapiens* donor sites, the 50% mixture with VAE-generated sequences reaches an F1-score of 0.9508, an MCC of 0.9013, and an AUROC of 0.9870 (baseline: F1-score 0.9371, MCC 0.8804, and AUROC 0.9860). Predictive performance degrades progressively as the real fraction decreases, with the largest drop at 10% real, most pronounced for the acceptor sites. These results indicate that the benefit of augmentation observed at 402 bp is preserved at the longer context, confirming that synthetic sequences remain an effective complement to real sequences for reducing real-sequence requirements.

**Table 13.** Scenario 3 (data augmentation) for the extended-context 2,002-bp sequences. Spliceator trained on mixtures of real and synthetic sequences and tested on real sequences for *Arabidopsis thaliana*, *Homo sapiens*, and *Danio rerio*. Baseline (100% real) shown in parentheses. SpliceRover is not included because its architecture is defined only for sequences up to 400 bp.

Species	Metric (baseline)	GAN			VAE			Diffusion		
		10%	25%	50%	10%	25%	50%	10%	25%	50%
Donor—Spliceator										
*Arabidopsis*	F1 (0.9378)	0.8816	0.9280	0.9327	0.8995	0.9252	0.9335	0.8738	0.9267	0.9352
	MCC (0.8801)	0.7868	0.8574	0.8670	0.8129	0.8526	0.8667	0.7731	0.8571	0.8692
	AUROC (0.9743)	0.9607	0.9669	0.9681	0.9620	0.9644	0.9674	0.9583	0.9665	0.9690
*Homo sapiens*	F1 (0.9371)	0.8953	0.9408	0.9475	0.9251	0.9450	0.9508	0.9197	0.9369	0.9480
	MCC (0.8804)	0.8138	0.8841	0.8954	0.8576	0.8913	0.9013	0.8498	0.8745	0.8958
	AUROC (0.9860)	0.9784	0.9845	0.9863	0.9807	0.9848	0.9870	0.9789	0.9802	0.9854
*Danio rerio*	F1 (0.9603)	0.9419	0.9532	0.9535	0.9320	0.9535	0.9574	0.9344	0.9506	0.9542
	MCC (0.9225)	0.8864	0.9079	0.9072	0.8713	0.9071	0.9151	0.8740	0.9014	0.9082
	AUROC (0.9858)	0.9755	0.9833	0.9831	0.9781	0.9824	0.9835	0.9761	0.9809	0.9827
Acceptor—Spliceator										
*Arabidopsis*	F1 (0.8990)	0.8184	0.9008	0.9256	0.8563	0.9174	0.9263	0.8420	0.9210	0.9302
	MCC (0.8180)	0.6933	0.8101	0.8498	0.7512	0.8386	0.8544	0.7281	0.8414	0.8597
	AUROC (0.9674)	0.9415	0.9624	0.9693	0.9593	0.9657	0.9700	0.9532	0.9653	0.9695
*Homo sapiens*	F1 (0.9089)	0.7723	0.8985	0.9123	0.8581	0.9005	0.9187	0.8532	0.8902	0.9171
	MCC (0.8286)	0.6588	0.8043	0.8244	0.7532	0.8148	0.8416	0.7413	0.7950	0.8340
	AUROC (0.9725)	0.9564	0.9629	0.9678	0.9576	0.9715	0.9741	0.9528	0.9636	0.9687
*Danio rerio*	F1 (0.9404)	0.8699	0.9335	0.9431	0.9022	0.9303	0.9412	0.9115	0.9390	0.9466
	MCC (0.8839)	0.7728	0.8723	0.8864	0.8218	0.8646	0.8824	0.8370	0.8778	0.8947
	AUROC (0.9769)	0.9640	0.9773	0.9792	0.9690	0.9732	0.9776	0.9711	0.9752	0.9797

GAN, generative adversarial network; VAE, variational autoencoder; MCC, Matthews correlation coefficient; AUROC, area under the receiver operating characteristic curve

## Discussion

Our evaluation of BlendSplice across 3 scenarios reveals that frequency blending substantially improves synthetic splice site quality. However, the mechanisms and magnitude of improvement vary substantially by model architecture and application context, providing insights into both the capabilities and limitations of current generative approaches for regulatory sequence synthesis.

### Frequency blending corrects model-specific weaknesses

The 3 generative models exhibit markedly different behaviors in response to frequency blending, revealing distinct failure modes and remediation pathways.

The GAN model shows the most remarkable improvement with blending. Without frequency priors, GAN-generated sequences exhibit systematic compositional biases, elevated GC content, and missing position-specific motifs critical for splice site function. This pattern reflects a fundamental property of adversarial training: the discriminator provides a global signal about sequence realism but does not explicitly enforce position-specific nucleotide constraints. Frequency blending effectively injects these local constraints, recovering purine-rich motifs upstream of donor sites (required for U1 snRNP recognition [41]) and pyrimidine-rich tracts upstream of acceptor sites (essential for U2AF binding [42]). This recovery is quantified by the PPT analysis, where blending raises the mean pyrimidine fraction and markedly reduces the malformation rate relative to unblended generation (for *H. sapiens*, from 26.7% to 1.1%), although the GAN tracts remain shorter than those of real sequences. The substantial functional improvements in the augmentation scenario confirm that these recovered features are biologically meaningful. The VAE model generates high-quality sequences even without blending, with frequency priors providing only marginal adjustments. Rather than contradicting the blending framework, this result supports the interpretation that architectures whose inductive biases naturally preserve positional structure may require little additional local calibration. The VAE therefore provides an informative reference point, demonstrating that frequency blending does not substantially distort sequences that are already well calibrated. This robustness reflects VAE’s learning objective: maximizing the evidence lower bound encourages structured latent representations that naturally capture position-dependent sequence features [23]. The convolutional architecture further supports hierarchical feature learning, with lower layers encoding local nucleotide preferences and higher layers capturing global composition patterns. The minimal benefit from blending suggests that VAE’s latent space inherently organizes splice site features in a biologically meaningful way. The diffusion model presents a more complex pattern. For *A. thaliana* and *D. rerio*, blending improves both direct biological metrics and functional performance as expected. However, for *H. sapiens*, blending systematically alters the sequence composition, yet maintains strong functional performance in downstream tasks. This overcorrection is directly visible in our quantitative analyses: the conservation heatmaps show that blended diffusion-generated sequences oversharpen the upstream rise at acceptor sites, and the PPT analysis shows that they generate pyrimidine runs longer than those of real sequences (for example, 10.81 versus 7.98 nucleotides for *H. sapiens*). This observation suggests that the diffusion model’s iterative refinement process [27] may overcorrect local sequence statistics while preserving higher-order dependencies that matter for biological function. Furthermore, species-specific behavior likely reflects differences in regulatory complexity: plant and fish splice sites exhibit relatively stereotyped consensus sequences, while mammalian splicing involves more distributed, context-dependent regulatory patterns [16]. Across all 3 models, the synthetic sequences were consistently less variable than real sequences: the GC content box plots and k-mer JSDs show narrower distributions that concentrate near the consensus. This indicates that the models reproduce the dominant splice site patterns well but underrepresent the tails of the real distribution, including atypical functional sites such as acceptors with weak or absent PPTs. Frequency blending improves fidelity to the dominant patterns rather than expanding the coverage of these atypical configurations, and the generation of rare and atypical functional sites remains an important direction for future work.

### Data augmentation outperforms complete sequence replacement

The stark contrast between scenario 2 (Train-Synthetic/Test-Real) and scenario 3 (augmentation) reveals fundamental limits to synthetic-sequence utility. Training exclusively on synthetic sequences produces models that transfer imperfectly to real test sequences, with the gap depending strongly on species: for *A. thaliana* and *D. rerio*, blended diffusion-generated sequences transfer reasonably well, whereas for *H. sapiens*, predictive performance degrades to near-random levels. This gap persists even with frequency blending, indicating that current generative models, regardless of the architecture, do not fully capture the diversity and complexity of natural splice sites, especially for the more complex mammalian sequences.

Several factors contribute to this gap in synthetic-to-real transferability. First, generative models trained on finite datasets tend to concentrate probability mass near the training distribution mode, underrepresenting extreme cases and rare variants. Our direct evaluation confirms this: synthetic sequences show narrower GC content distributions and higher k-mer JSDs in the tails compared to real sequences. Second, frequency priors computed from training data necessarily reflect only observed patterns and cannot generalize to unobserved or underrepresented sequence contexts. Third, some biological features may involve long-range dependencies or complex combinatorial interactions that current architectures fail to capture fully.

In contrast, augmenting 50% real training sequences with 50% synthetic sequences achieves near-baseline predictive performance across all 3 species, both splice categories, and all models. This finding establishes data augmentation as BlendSplice’s optimal application: halving real-sequence requirements while maintaining state-of-the-art predictive performance. This 50% real/50% synthetic ratio represents the minimal real-sequence fraction needed to anchor learning effectively: lower real-sequence proportions (augmented with correspondingly higher synthetic proportions) show progressive predictive performance degradation, particularly at 10% real sequences. The practical value of augmentation therefore lies in data-scarce settings rather than in surpassing a model trained on abundant real sequences: when real sequences are limited, augmentation with synthetic sequences substantially recovers predictive performance, and the 50% real/50% synthetic result shows that the real-sequence requirement can be halved without loss of predictive performance.

The success of augmentation despite the weaker predictive performance of complete replacement suggests that real sequences provide critical “ground truth” examples that calibrate model learning beyond what frequency-blended synthetic sequences can achieve. Real sequences likely cover rare variants, edge cases, and complex motif combinations that generative models systematically miss, while synthetic sequences add volume and potentially reduce sampling bias in limited real datasets.

### Contrast between direct and indirect evaluation

Scenario 1 demonstrates that splice site predictors trained on real sequences successfully recognize frequency-blended synthetic sequences, achieving near-baseline predictive performance across all models and species. This contrasts with scenario 2, where predictors trained on synthetic sequences transfer only partially to real sequences and fail almost entirely for *H. sapiens*. This contrast (synthetic sequences can fool models trained on real sequences, but models trained on synthetic sequences generalize only partially to real sequences) reveals an important distinction between capturing discriminative patterns and capturing the full distribution.

Frequency-blended synthetic sequences successfully preserve the position-specific nucleotide preferences, motif combinations, and local sequence contexts that trained predictors use for splice site recognition. However, synthetic sequences used for training lack the distributional breadth, rare variant coverage, and complex feature interactions that are present in real sequences. A predictor trained on real sequences can recognize synthetic sequences (because synthetic sequences contain patterns that are also present in real sequences) but cannot fully learn to recognize the full range of real patterns from synthetic sequences (because synthetic sequences underrepresent that range).

This observation has implications for how we evaluate generative models for biological sequences. Traditional distributional metrics (GC content or nucleotide conservation scores) measure statistical similarity but may not capture functional features that determine biological activity. Our k-mer JSD analysis quantifies this statistical similarity but, like the other direct metrics, does not by itself establish functional adequacy. The diffusion model’s behavior on *H. sapiens*, where blending alters composition yet maintains functional performance, exemplifies this disconnect. Functional evaluation through downstream tasks provides complementary information about whether generated sequences preserve biologically meaningful patterns, even when statistical properties diverge from training data.

The independent SpliceAI validation reinforces this point: an external model that shares neither architecture nor training data with the proxy classifiers recognizes the blended synthetic sequences at rates approaching the real-sequence reference, indicating that the high proxy predictive performance reflects genuine biological signal rather than feature overlap specific to SpliceRover and Spliceator.

### Species and site type differences reflect biological complexity

Consistent patterns emerge across all scenarios: *A. thaliana* and *D. rerio* sequences are easier to generate than *H. sapiens* ones, and donor sites are easier than acceptor sites [50]. These differences are consistent with reported divergence in splice site sequence composition across plant and animal lineages [51] and with the scaling of alternative-splicing complexity with organismal complexity [52].

Plant and fish splice sites exhibit relatively conserved consensus sequences with limited positional variation, making them more amenable to generative modeling. Mammalian splicing involves weaker conservation and more distributed regulatory information, where auxiliary sequence elements modulate splice site strength in context-dependent ways [16]. The greater challenge in generating human sequences reflects this fundamental complexity: models must capture not just local motifs but also their interactions and context-dependent modulation, consistent with the larger synthetic-to-real transfer gap observed for *H. sapiens* in scenario 2.

Acceptor sites prove consistently harder than donor sites across all models. This difficulty reflects genuine biological complexity: acceptor recognition requires variable-length PPTs, branch point sequences at variable distances, and combinatorial interactions among these elements. Our quantitative analysis confirms that the models struggle with PPT generation: without blending, GAN- and diffusion-generated tracts are pyrimidine-poor with elevated malformation rates, and although blending raises pyrimidine content and lowers malformation rates, the diffusion model overshoots tract integrity, producing pyrimidine runs longer than those of real sequences. This suggests that current architectures, which process sequences with fixed-size local receptive fields or attention windows, may be insufficient for faithfully capturing the variable-length regulatory regions characteristic of acceptor sites.

The persistence of these difficulties across all 3 species, and their concentration at acceptor sites, indicates that they reflect intrinsic biological complexity rather than artifacts of any single dataset and points to architectures with adaptive or longer-range receptive fields as a promising direction for better modeling of variable-length regulatory elements.

## Conclusions

We introduced BlendSplice, a posttraining frequency-blending technique that enhances synthetic splice site quality across diverse generative architectures. By linearly combining model predictions with position-specific nucleotide priors extracted from real sequences, frequency blending provides a simple, posttraining, model-agnostic mechanism that improves biological realism and functional utility for architectures prone to compositional bias and that is harmless for architectures that naturally learn positional constraints.

Our evaluation across 3 generative models (GAN, VAE, and diffusion models), 3 species (*A. thaliana*, *H. sapiens*, and *D. rerio*), 2 splice site types (donor and acceptor), and 2 sequence lengths (402 and 2,002 bp) reveals that blending at λ=0.5 consistently improves the balance between learned generative patterns and empirical sequence constraints. However, the mechanism and magnitude of improvement varies by architecture: GAN benefits most from blending to correct compositional biases, VAE generates high-quality sequences even without blending, and the diffusion model exhibits species-dependent behavior reflecting differences in regulatory complexity. These patterns were consistent across both sequence lengths and were corroborated by quantitative k-mer divergence analysis and by independent validation with SpliceAI.

The most important finding is that augmenting 50% real sequences with 50% synthetic sequences achieves near-baseline predictive performance across all experimental conditions. This establishes a practical path to reduce real genomic data requirements in machine learning applications while maintaining state-of-the-art predictive performance. In contrast, training exclusively on synthetic sequences transfers only partially to real test sequences, with the largest gap observed for *H. sapiens*, indicating limits to the use of synthetic sequences as a complete replacement for authentic genomic sequences.

These results have implications beyond splice site generation. The success of frequency blending suggests that hybrid approaches that combine learned representations with domain-specific priors may be broadly applicable to other regulatory sequence classes. The contrast between direct biological metrics and functional performance highlights the need for multifaceted evaluation frameworks that assess both distributional fidelity and biological activity. The data augmentation findings provide a template for privacy-conscious genomic data sharing, where reduced real data exposure can be achieved without sacrificing predictive performance.

Future work should address current limitations in several directions. First, extending frequency blending to capture longer-range dependencies beyond immediate neighbors could better model the distributed regulatory elements characteristic of mammalian sequences. Relatedly, improving the coverage of atypical functional sites, such as acceptors with weak or absent PPTs, would address the narrower distributions observed for synthetic sequences. Second, applying BlendSplice to other regulatory elements (enhancers, promoters, and transcription factor binding sites) would test the generalizability of frequency blending across sequence classes with different organizational principles. Third, combining frequency blending with privacy-preserving training methods, such as differential privacy or federated learning, could enable genomic data sharing with formal privacy protections beyond the practical benefit of using fewer real data. Finally, experimental validation through *in vitro* splicing assays or RNA sequencing would provide definitive evidence that computationally high-quality synthetic sequences are functionally recognized by the spliceosome and exhibit expected splicing activity in biological systems.

## Data Availability

The 402-bp datasets for *A. thaliana* and *H. sapiens* used to train the generative models can be downloaded from https://github.com/XueyanLiu-creator/DRANetSplicer/tree/main/data/dna_sequences, and the data for *A. thaliana* and *H. sapiens* used for indirect evaluation can be downloaded from https://github.com/OluwadareLab/EnsembleSplice/tree/main/ENSdatasets. The extended-context (2,002-bp) datasets for all 3 species (*A. thaliana*, *H. sapiens*, and *D. rerio*), together with all code required to reproduce the experiments, are available at https://github.com/EspoirKabanga/BlendSplice. The synthetic sequences generated for each sequence length, generative model, and value of λ are also made available in the same repository.
