# Supplementary material for: BlendSplice: A Frequency-Blended Generative Framework for In Silico Synthesis of Biologically Realistic Splice Site Sequences
Source: Comput Struct Biotechnol J. 2026 Jul 23;35(1):0171. doi: 10.34133/csbj.0171 (PMC13392282; doi:10.34133/csbj.0171)
Supplement: Supplementary 1 — Supplementary Text Figs. S1 to S22 Tables S1 to S3 [file csbj.0171.f1.pdf]

Supplementary Materials for: BlendSplice: A Frequency-Blended Generative Framework for In Silico Synthesis of Biologically Realistic Splice Site Sequences

Espoir Kabanga[1,2], Seonil Jee[1], Arnout Van Messem[3], and Wesley De Neve[1,2]

[1] Center for Biosystems and Biotech Data Science, Ghent University Global Campus, Incheon, South Korea
{espoir.kabanga, seonil.jee, wesley.deneve}@ghent.ac.kr
[2] IDLab, Department of Electronics and Information Systems, Ghent University, Ghent, Belgium
[3] Department of Mathematics, University of Liège, Liège, Belgium
arnout.vanmessem@uliege.be

Extended Lambda Analysis

In the main manuscript, we introduced BlendSplice's frequency blending mechanism, which linearly combines model-learned probability distributions with empirical position-specific nucleotide frequencies extracted from real training sequences. The blending weight λ controls the relative contribution of these two sources of information according to:

$$\mathbf{p}_{\text{blend}}(i) = (1-\lambda) \cdot \mathbf{p}_{\text{model}}(i) + \lambda \cdot \mathbf{p}_{\text{freq}}(i) \tag{1}$$

where $p_{\text{model}}(i)$ represents the probability distribution predicted by the generative model at position i, and $p_{\text{freq}}(i)$ denotes the empirical frequency prior. The main manuscript reported results for $\lambda = 0.0$ (*No-Blend*), representing pure model predictions without frequency guidance, and $\lambda = 0.5$ (*Blend*), representing balanced integration of model probabilities and empirical priors with equal weighting.

To provide comprehensive understanding of the model-prior trade-off, we conducted additional experiments at intermediate lambda values: $\lambda = 0.25$ and $\lambda = 0.75$. At $\lambda = 0.25$, model probabilities contribute 75% while frequency priors contribute 25%, representing *model-dominant blending* where generative diversity is prioritized with light constraint enforcement. Conversely, at $\lambda = 0.75$, frequency priors contribute 75% while model probabilities contribute only 25%, representing *prior-dominant blending* where biological constraints are strongly enforced with reduced model autonomy. These configurations enable systematic exploration of the spectrum between pure generative output ($\lambda = 0.0$) and the balanced approach ($\lambda = 0.5$) validated in the main results.

In what follows, we first present direct evaluation metrics examining the statistical properties of generated sequences, including `GC` content distribution, 3-mer context preservation, position-specific nucleotide conservation, and sequence logo analysis. Subsequently, we report indirect functional validation through a state-of-the-art splice site classifier, SpliceRover, for both species (*Arabidopsis thaliana* and *Homo sapiens*) and splice site types (donor and acceptor), enabling comprehensive assessment of how frequency blending influences synthetic sequence quality across the entire lambda spectrum.

Direct evaluation

`GC` content: Figures 1, 2, and 3 present `GC` content distributions for synthetic sequences generated at $\lambda \in \{0.0, 0.25, 0.5, 0.75\}$ compared to real genomic sequences, with vertical dashed lines indicating mean values. For the GAN model (Fig. 1), sequences generated with $\lambda = 0.75$ exhibit mean `GC` content closely aligned with real

data across all conditions. In contrast, at $\lambda = 0.25$, the mean GC content is elevated relative to the real reference, with this deviation being particularly pronounced for *Arabidopsis* donor and acceptor sequences. The VAE model (Fig. 2) demonstrates remarkable consistency, with mean GC content remaining similar across all lambda values and datasets, indicating that VAE inherently captures compositional constraints independently of frequency prior strength. For the diffusion model (Fig. 3), at $\lambda = 0.75$, the mean GC content is lower compared to the other lambda values ($\lambda \in \{0.0, 0.25, 0.5\}$), all of which exhibit minimal deviation from the real data. These observations reveal distinct model-specific responses to frequency blending: the GAN model achieves optimal GC content alignment with real data at higher λ, the VAE model maintains robustness across the entire spectrum, and the diffusion model maintains close GC content match to real data at $\lambda \in \{0.0, 0.25, 0.5\}$ but shows lower GC content than real data at $\lambda = 0.75$.

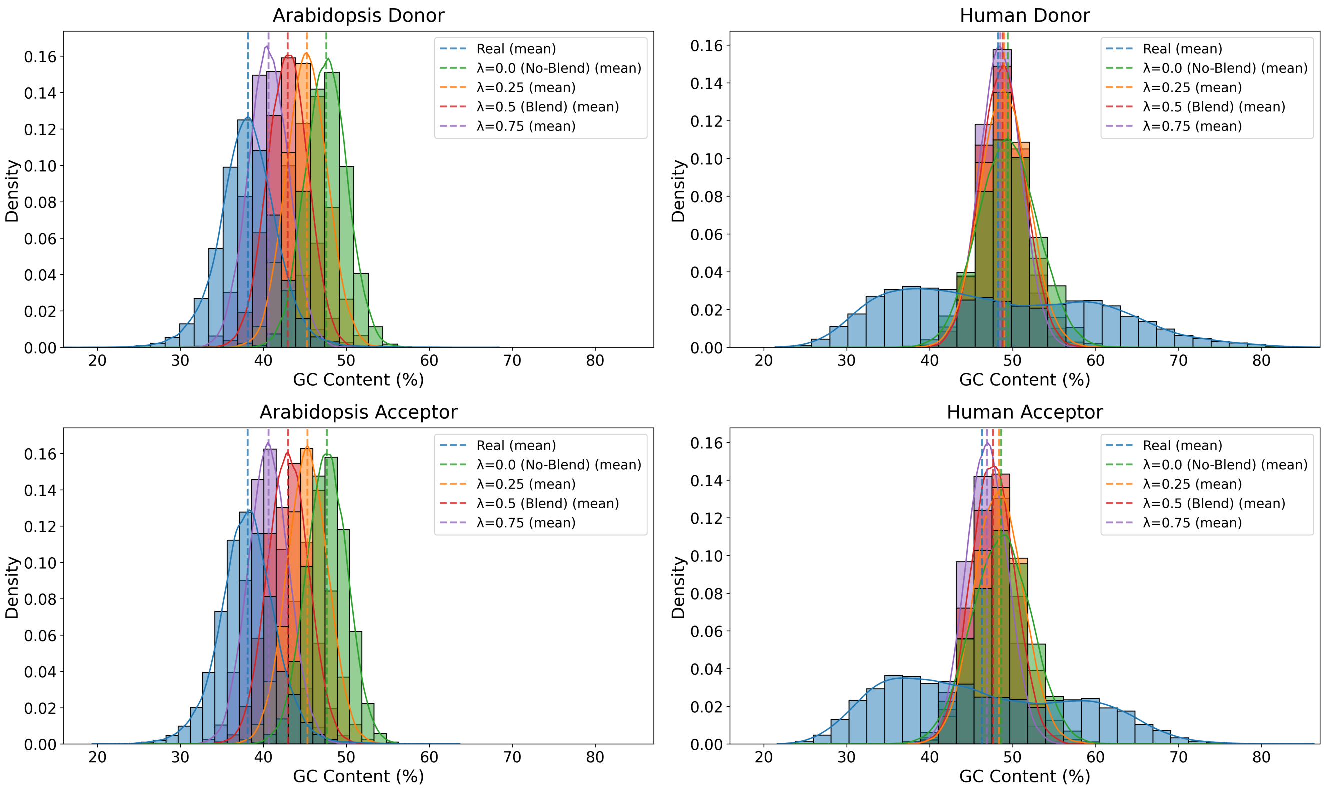

Fig. 1: GC content for the GAN model.

3-mer context: Figures 4, 5, and 6 present the frequencies of the top-5 most frequent 3-mers in the immediate upstream region (left of dashed line) and the top-5 in the immediate downstream region (right of dashed line) of the splice site for synthetic sequences generated at $\lambda \in \{0.0, 0.25, 0.5, 0.75\}$ compared to real genomic sequences. For the GAN model (Fig. 4), at $\lambda = 0.25$, the 3-mer frequencies show moderate alignment with real data, though several motifs exhibit noticeable deviations. At $\lambda = 0.75$, GAN-generated sequences achieve substantially better

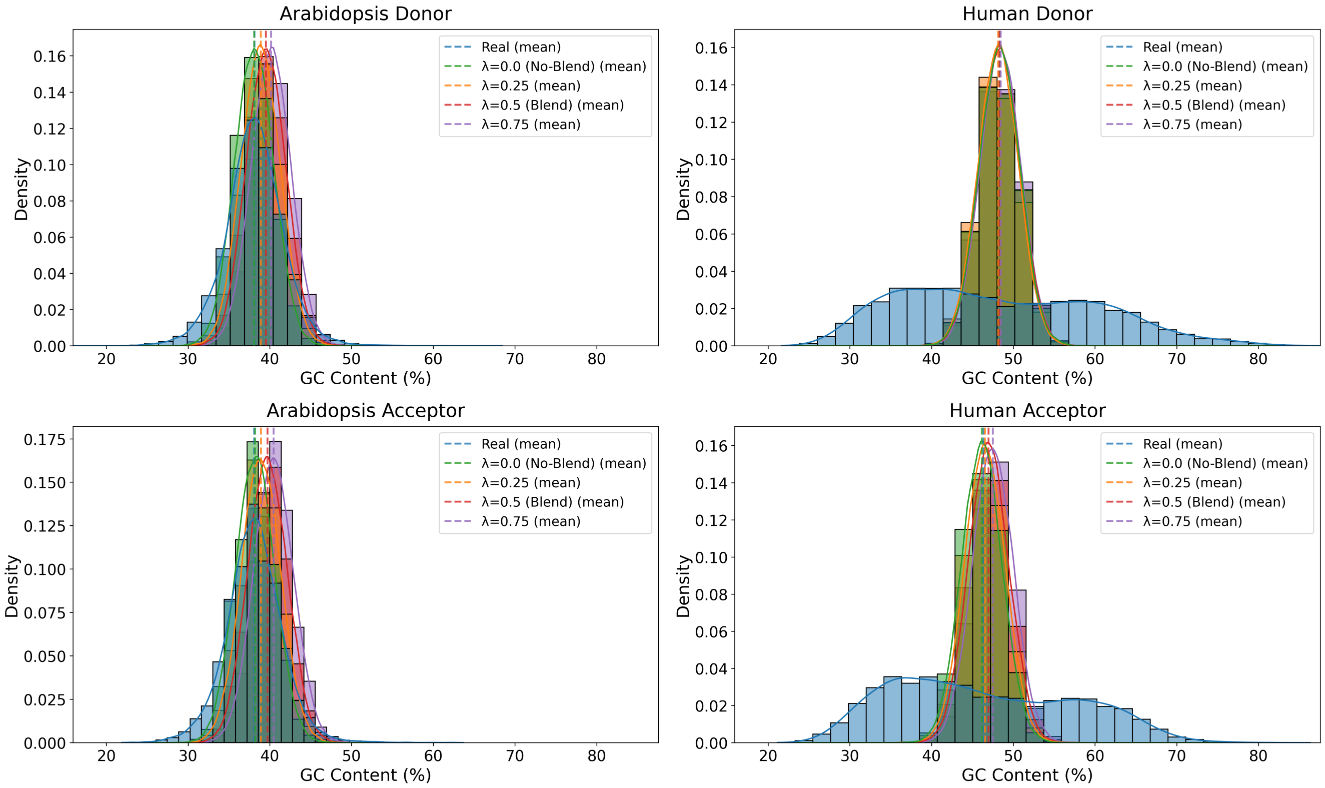

Fig. 2: `GC` content for the VAE model.

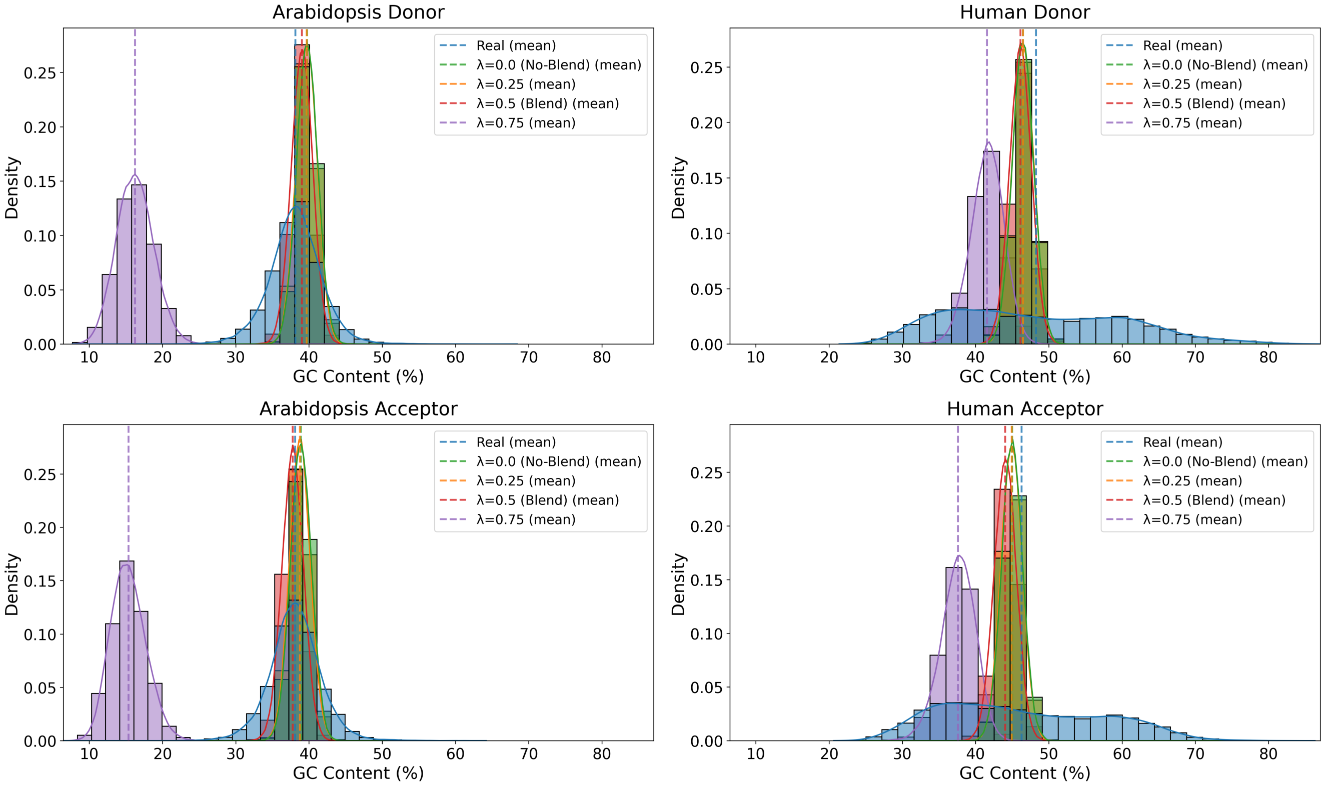

Fig. 3: `GC` content for the diffusion model.

3-mer frequency matching across all datasets, with most motifs closely approximating real distributions. The VAE model (Fig. 5) demonstrates remarkable consistency across all lambda values. The 3-mer frequencies at $\lambda = 0.25$ are nearly identical to those at $\lambda = 0.5$ and $\lambda = 0.75$, all closely matching real data. This indicates that VAE inherently captures local sequence context independently of frequency prior strength. For the diffusion model (Fig. 6), a striking pattern emerges: at $\lambda = 0.75$, specific 3-mers become dramatically overrepresented, with frequencies approaching 1.0 for motifs such as `AAG` in donor sites and `GTT` in acceptor sites. In contrast, $\lambda \in \{0.0, 0.25, 0.5\}$ maintain more balanced 3-mer distributions, though still exhibiting elevated frequencies for certain motifs compared to real data. This suggests that excessive frequency prior enforcement ($\lambda = 0.75$) causes the diffusion model to over-concentrate on dominant motifs at the expense of distributional diversity.

In summary, our results demonstrate that the GAN model benefits from higher λ for 3-mer context preservation, the VAE model maintains robust local context across the lambda spectrum, while the diffusion model shows overfitting to dominant motifs at $\lambda = 0.75$.

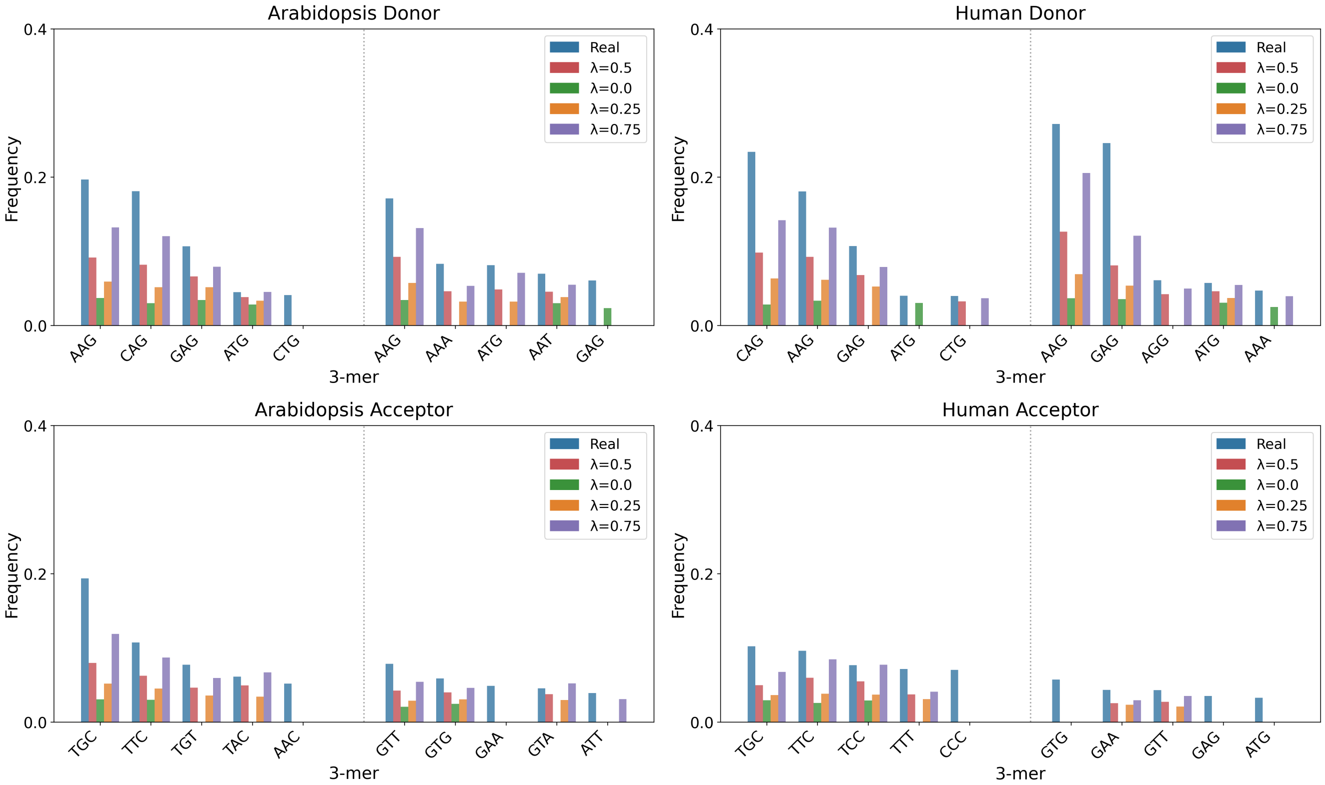

Fig. 4: 3-mer context for the GAN model.

Nucleotide conservation: Figures 7, 8, and 9 present position-specific nucleotide conservation scores for synthetic sequences generated at $\lambda \in \{0.0, 0.25,$

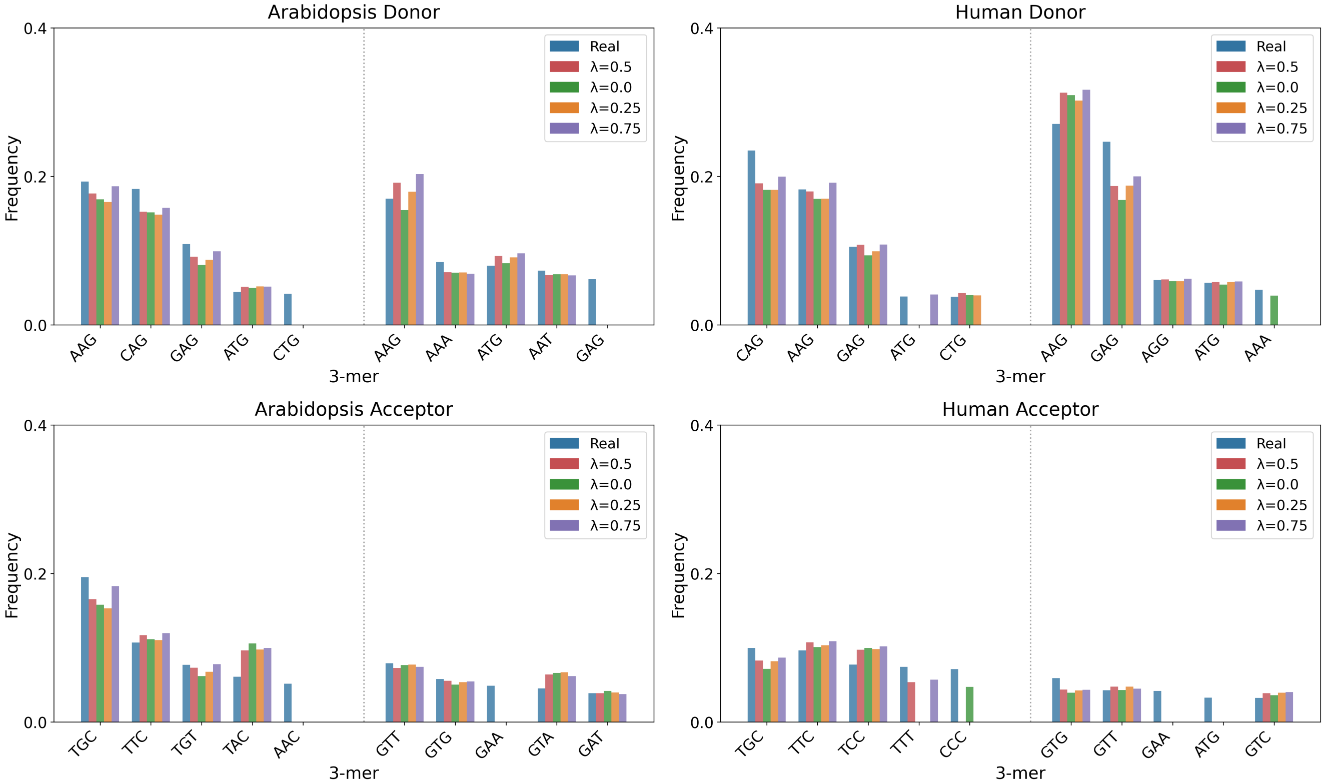

Fig. 5: 3-mer context for the VAE model.

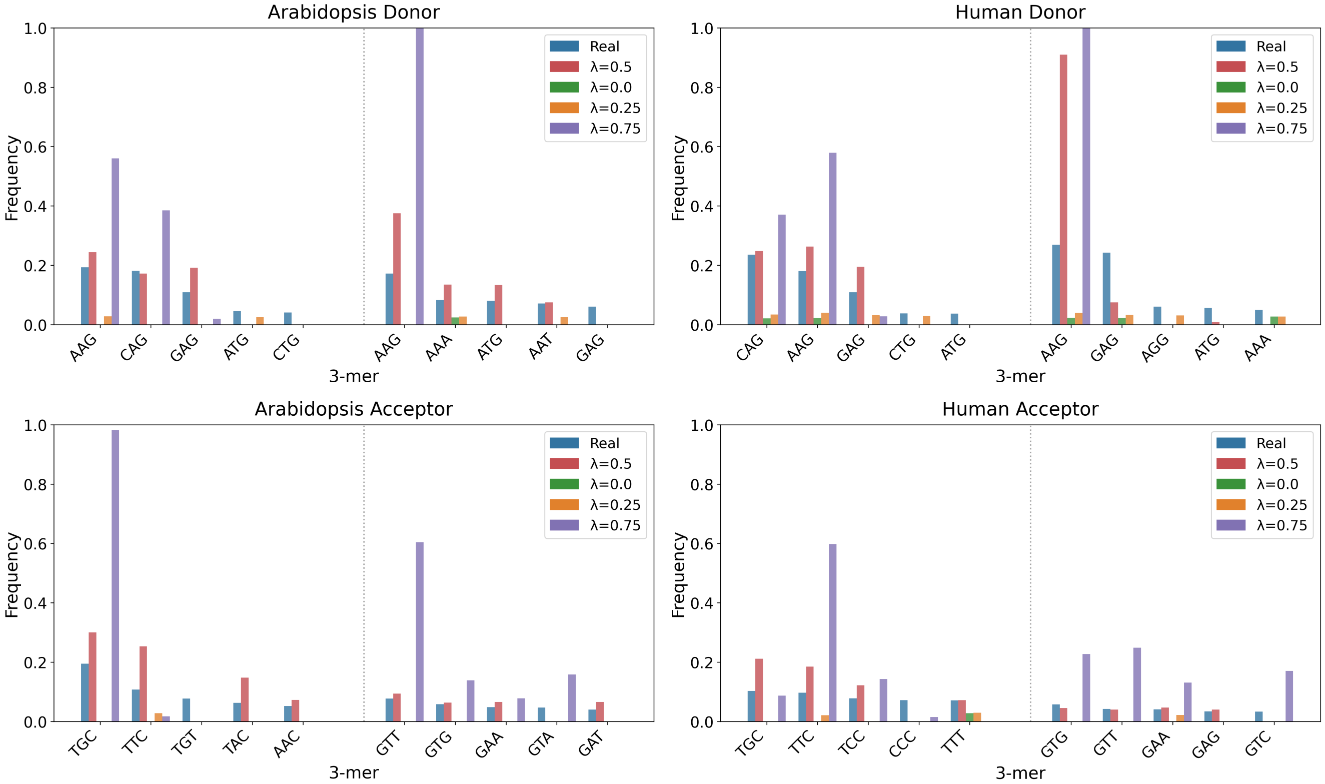

Fig. 6: 3-mer context for the diffusion model.

$0.5, 0.75\}$ compared to real genomic sequences. The conservation score at each position reflects the degree of nucleotide preference consistency across the sequence ensemble, with higher values indicating stronger positional constraints. The splice junction typically exhibits peak conservation due to canonical dinucleotide requirements. For the GAN model (Fig. 7), both $\lambda = 0.25$ and $\lambda = 0.75$ capture the characteristic conservation peak at the splice junction. At $\lambda = 0.75$, conservation at the junction more closely matches the real data, with conservation patterns in flanking regions also showing improved alignment. At $\lambda = 0.25$, while junction conservation is preserved, flanking region patterns exhibit greater deviation from real data, particularly in acceptor sites where upstream conservation profiles are less well-matched. The VAE model (Fig. 8) demonstrates remarkable consistency across all lambda values. Conservation profiles at $\lambda = 0.25$ are nearly indistinguishable from those at $\lambda = 0.75$ and $\lambda = 0.5$, all closely tracking the real data patterns. The sharp conservation peaks at splice junctions and the moderate conservation in flanking regions are faithfully reproduced regardless of frequency prior strength, confirming VAE's inherent capture of position-specific constraints. For the diffusion model (Fig. 9), $\lambda = 0.75$ produces dramatically elevated conservation scores across extended regions flanking the splice junction, particularly visible in downstream donor regions and upstream acceptor regions where conservation approaches 1.0. This over-conservation indicates that strong frequency prior enforcement causes the diffusion model to generate near-uniform sequences at many positions, sacrificing distributional diversity. In contrast, $\lambda \in \{0.0, 0.25, 0.5\}$ maintain more realistic conservation patterns, though $\lambda = 0.25$ and $\lambda = 0.5$ better capture the junction peaks compared to $\lambda = 0.0$.

In summary, our results demonstrate that the GAN model benefits from higher λ for improved conservation profile matching, the VAE model maintains accurate conservation patterns across all lambda values, and the diffusion model shows over-fitting at $\lambda = 0.75$, with intermediate values providing more balanced conservation profiles.

Sequence logos: Figures 10, 11, and 12 present sequence logos comparing real splice sites with synthetic sequences generated at $\lambda \in \{0.0, 0.25, 0.5, 0.75\}$. Each row displays position-specific nucleotide frequencies, with letter height representing the frequency of each base at that position. For the GAN model (Fig. 10), at $\lambda = 0.25$, the canonical splice junction dinucleotides (`GT` for donor, `AG` for acceptor) are present but flanking regions exhibit noticeable deviations from real patterns. At $\lambda = 0.75$, flanking regions show improved alignment with real data, including more defined polypyrimidine-like stretches downstream of donor sites and upstream of acceptor sites. The increased frequency prior weight improves the characteristic compositional biases in adjacent regions. The VAE model (Fig. 11) demonstrates remarkable consistency across all lambda values. Sequence logos at $\lambda = 0.25$ are nearly identical to those at $\lambda = 0.5$ and $\lambda = 0.75$, all closely matching real data patterns. The canonical `GT`/`AG` dinucleotides are preserved with appropriate frequency, and flanking region motifs maintain realistic diversity regardless of frequency prior strength. This stability confirms that VAE's latent representations inherently capture position-specific nucleotide preferences without requiring explicit frequency guidance. For the diffusion model (Fig. 12), $\lambda = 0.75$ produces sequence logos with dramatically reduced diversity, displaying near-uniform nucleotide composition at many positions. Downstream donor regions become almost entirely thymine-rich, and upstream acceptor regions show extreme cytosine dominance, indicating severe over-fitting to dominant frequency patterns. The letters in these regions reach maximum height (frequency ≈ 1.0), reflecting the loss of distributional diversity observed in previous metrics. In contrast, $\lambda = 0.25$ and $\lambda = 0.5$ maintain more realistic sequence diversity while preserving junction motifs, with $\lambda = 0.5$ providing the best balance between motif preservation and sequence variability.

In summary, our sequence logo comparisons reinforce the model-specific sensitivity patterns: the GAN model achieves improved motif fidelity at higher λ values, the VAE model maintains robust logo profiles across the entire lambda spectrum, and the diffusion model exhibits severe over-fitting at $\lambda = 0.75$, with intermediate values yielding more biologically realistic sequence diversity.

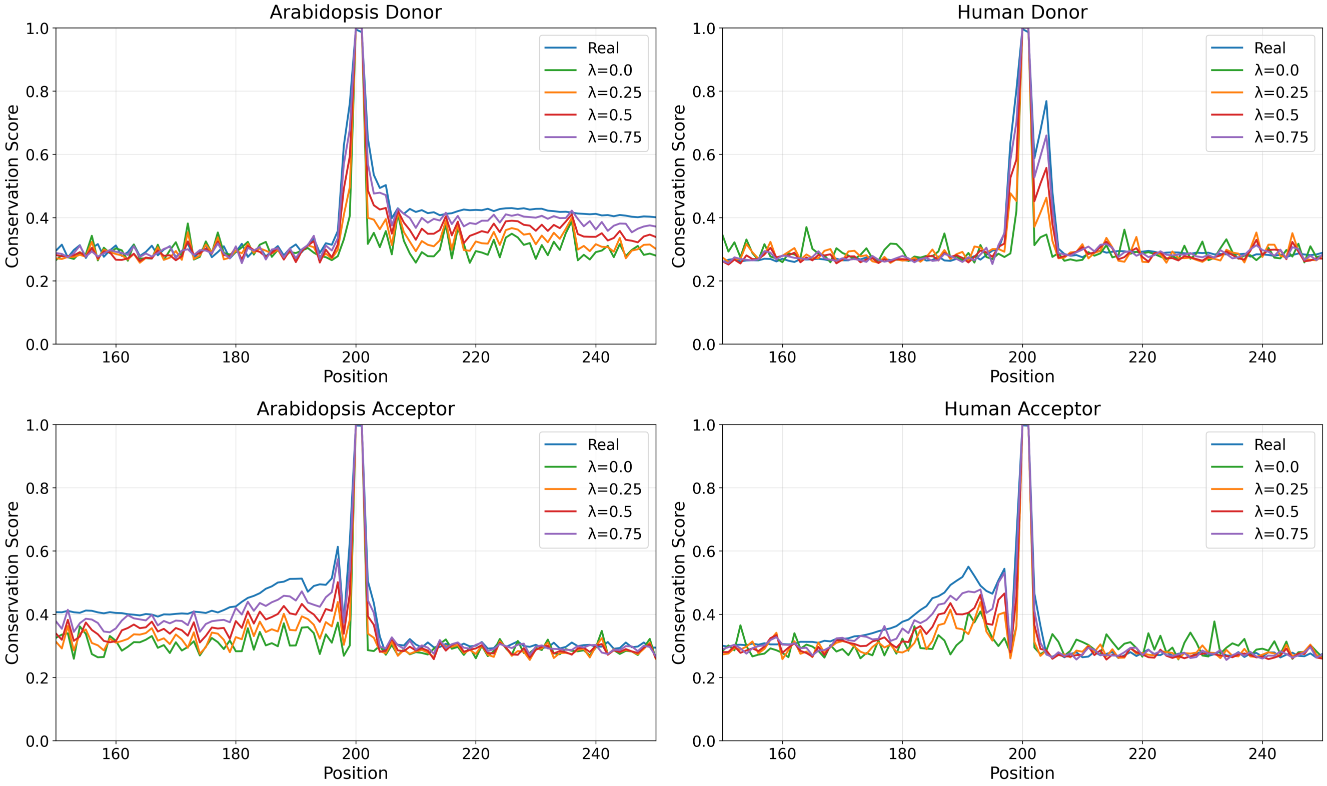

Fig. 7: Nucleotide conservation scores for the GAN model.

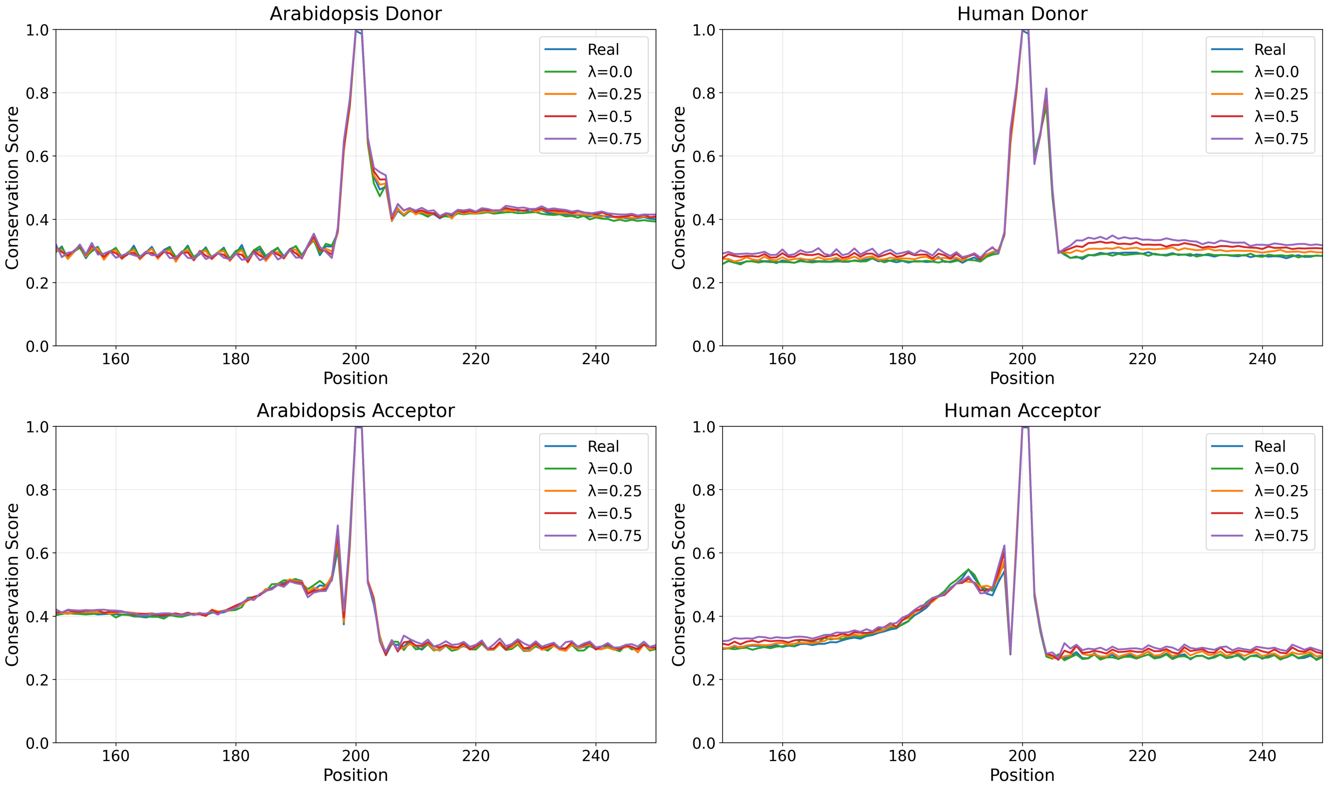

Fig. 8: Nucleotide conservation scores for the VAE model.

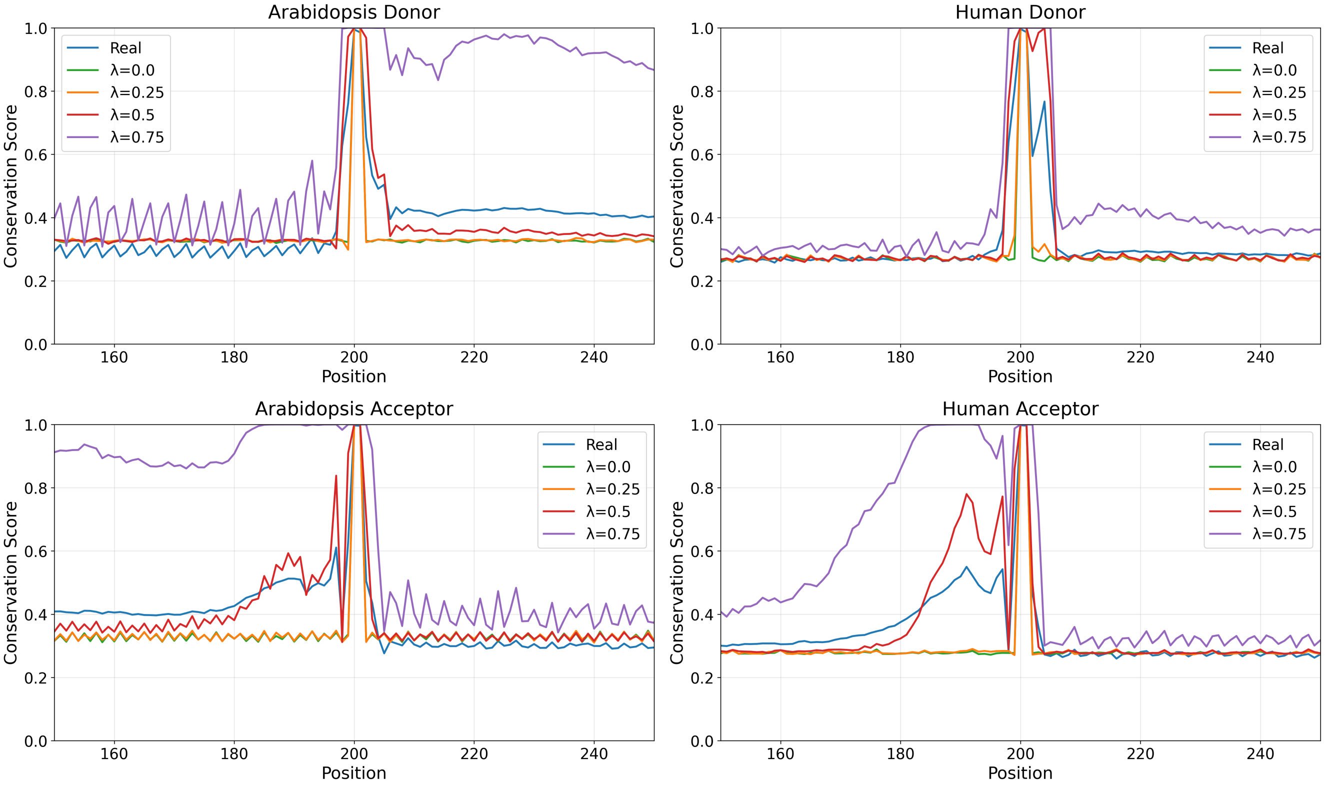

Fig. 9: Nucleotide conservation scores for the diffusion model.

Indirect evaluation

Table 1 describes the characteristics of SpliceRover [1] and Spliceator [2] models

Table 1: Characteristics of the SpliceRover and Spliceator model architectures.

Layer	SpliceRover	Spliceator
**1**	Conv 70 x (9, 4)	Conv 16 x (7, 4)
**2**	Dropout ($p = 0.2$)	MaxPool (2, 1)
**3**	Conv 100 x (7, 1)	Dropout ($p = 0.2$)
**4**	Dropout ($p = 0.2$)	Conv 32 x (6, 1)
**5**	Conv 100 x (7, 1)	MaxPool (2, 1)
**6**	MaxPool (3, 1)	Dropout ($p = 0.2$)
**7**	Dropout ($p = 0.2$)	Conv 64 x (6, 1)
**8**	Conv 200 x (7, 1)	MaxPool (2, 1)
**9**	MaxPool (4, 1)	Dropout ($p = 0.2$)
**10**	Dropout ($p = 0.2$)	Flatten
**11**	Conv 250 x (7, 1)	Dense 100 (ReLU)
**12**	MaxPool (4, 1)	Dense 2 (Softmax)
**13**	Dropout ($p = 0.2$)	-
**14**	Flatten	-
**15**	Dense 512 (ReLU)	-
**16**	Dropout ($p = 0.2$)	-
**17**	Dense 2 (Softmax)	-

We converted the DNA sequences into a numerical representation using one-hot encoding, where each nucleotide is represented as follows:

$$\text{A} = \begin{bmatrix}1\\0\\0\\0\end{bmatrix}, \quad \text{C} = \begin{bmatrix}0\\1\\0\\0\end{bmatrix}, \quad \text{G} = \begin{bmatrix}0\\0\\1\\0\end{bmatrix}, \quad \text{T} = \begin{bmatrix}0\\0\\0\\1\end{bmatrix}, \quad \text{N} = \begin{bmatrix}0\\0\\0\\0\end{bmatrix}$$

For training the models, we adopted a standardized approach to hyperparameter selection to ensure comparability across the two splice site classifiers. This involved utilizing the Adam optimizer [3] with a learning rate of 0.001, the categorical cross-entropy loss function, and a batch size of 64. The Adam optimizer was selected for its effectiveness in handling sparse gradients and its adaptive learning rate properties. The learning rate of 0.001 balances fast convergence with training stability across diverse tasks and datasets. The uniform application of these hyperparameters across all models facilitated fair comparison and removed potential biases from different training conditions.

To prevent overfitting, we employed early stopping with a maximum of 30 epochs and patience of 5, terminating training if no improvement in validation loss was observed after 5 consecutive epochs.

Scenario 1 Table 2 presents proxy evaluation results using SpliceRover, a state-of-the-art splice site classifier trained on real genomic sequences and tested on synthetic sequences generated at $\lambda \in \{0.0, 0.25, 0.5, 0.75\}$. High predictive performance indicates that synthetic sequences preserve the discriminative features characteristic of

functional splice sites. The baseline (Real) represents the upper bound predictive performance when testing on authentic genomic sequences.

GAN model performance The GAN model exhibits strong lambda-dependent predictive performance. At $\lambda = 0.0$ (*No-Blend*), F1-scores are extremely low (0.0538–0.2143), with AUROC values ranging from 0.6036 to 0.7979, indicating that pure GAN-generated sequences poorly capture splice site characteristics. At $\lambda = 0.25$, predictive performance improves substantially, with F1-scores increasing to 0.3253–0.5693 and AUROC to 0.8062–0.8821, demonstrating that even modest frequency prior incorporation enhances biological realism. Further improvement occurs at $\lambda = 0.5$, where F1-scores reach 0.5842–0.7414 and AUROC 0.8416–0.9165. At $\lambda = 0.75$, GAN achieves its best predictive performance with F1-scores of 0.7114–0.8525 and AUROC of 0.9113–0.9539, representing 74–89% of baseline F1-score performance. This monotonic improvement across the lambda spectrum confirms that GAN benefits substantially from frequency prior guidance.

VAE model performance The VAE model demonstrates remarkable robustness across lambda values. Even at $\lambda = 0.0$, the VAE model achieves strong predictive performance with F1-scores of 0.8391–0.9118 and AUROC of 0.9365–0.9750, representing 87–95% of baseline performance. At $\lambda = 0.25$, predictive performance remains nearly identical (F1: 0.8449–0.8965, AUROC: 0.9549–0.9783), with only marginal changes. Predictive performance at $\lambda = 0.5$ and $\lambda = 0.75$ similarly shows minimal variation (F1: 0.8621–0.9294, AUROC: 0.9578–0.9840), consistently achieving 90–97% of baseline F1-scores. The tight clustering of performance metrics across all lambda values indicates that VAE's latent space organization inherently captures the discriminative features necessary for splice site recognition, requiring minimal explicit frequency guidance.

Diffusion model performance The diffusion model exhibits the most remarkable lambda sensitivity. At $\lambda = 0.0$, predictive performance is poor (F1: 0.1296–0.2501, AUROC: 0.5992–0.6829), comparable to near-random classification in some cases. At $\lambda = 0.25$, improvement is modest (F1: 0.1485–0.2929, AUROC: 0.5937–0.7289), with predictive performance remaining substantially below other models. However, at $\lambda = 0.5$, a notable improvement emerges, with F1-scores jumping to 0.8201–0.9638 and AUROC to 0.9370–0.9925, achieving 85–100% of baseline performance. At $\lambda = 0.75$, the diffusion model reaches near-perfect predictive performance (F1: 0.9673–0.9814, AUROC: 0.9987–0.9998), in several cases *exceeding baseline performance* (e.g., Human Donor F1: 0.9791 vs. baseline 0.9600). This dramatic non-linear improvement from $\lambda = 0.25$ to $\lambda = 0.5$ and further to $\lambda = 0.75$ reveals that the diffusion model requires substantial frequency prior strength ($\lambda \geq 0.5$) to generate biologically realistic splice site sequences.

Model comparison and optimal lambda Our results reveal distinct architectural dependencies on frequency blending. The GAN model shows continuous improvement with increasing λ, with $\lambda = 0.75$ yielding optimal predictive performance. The VAE model maintains consistently strong predictive performance across the entire lambda spectrum, demonstrating architectural robustness. The diffusion model shows threshold-like behavior, requiring $\lambda \geq 0.5$ for acceptable predictive performance, with $\lambda = 0.75$ producing near-perfect synthetic sequences. The choice of $\lambda = 0.5$ in the main text represents a balanced compromise: it enables strong diffusion model performance while maintaining diversity in GAN-generated sequences and preserving VAE's inherent capabilities. For applications prioritizing maximum biological realism over generative diversity, $\lambda = 0.75$ provides superior predictive performance, particularly for the diffusion model.

Scenario 2 Table 3 presents proxy evaluation results for the inverse scenario: SpliceRover trained on synthetic sequences and tested on real genomic sequences. This scenario evaluates synthetic sequence transferability, which is

Table 2: Proxy evaluation results for SpliceRover: *Train-Real/Test-Synthetic*

Model	Dataset	Metric	Real	$\lambda = 0.0$	$\lambda = 0.25$	$\lambda = 0.5$	$\lambda = 0.75$
GAN	*Arabidopsis* Donor	F1-score	0.9595	0.0538	0.4675	0.6075	0.8158
		AUROC	0.9899	0.6036	0.8391	0.8416	0.9448
	Arabidopsis Acceptor	F1-score	0.9573	0.1206	0.3253	0.6328	0.7114
		AUROC	0.9904	0.6829	0.8062	0.8604	0.9132
	Human Donor	F1-score	0.9600	0.1808	0.5693	0.7414	0.8325
		AUROC	0.9916	0.7668	0.8821	0.9165	0.9539
	Human Acceptor	F1-score	0.9591	0.2143	0.4328	0.5842	0.7215
		AUROC	0.9890	0.7979	0.8137	0.8955	0.9113
VAE	*Arabidopsis* Donor	F1-score	0.9595	0.8395	0.8965	0.8858	0.9249
		AUROC	0.9899	0.9365	0.9719	0.9578	0.9815
	Arabidopsis Acceptor	F1-score	0.9573	0.8687	0.8467	0.8958	0.8744
		AUROC	0.9904	0.9495	0.9566	0.9641	0.9682
	Human Donor	F1-score	0.9600	0.9118	0.9139	0.9294	0.9309
		AUROC	0.9916	0.9750	0.9783	0.9817	0.9840
	Human Acceptor	F1-score	0.9591	0.8391	0.8449	0.8621	0.8737
		AUROC	0.9890	0.9628	0.9549	0.9679	0.9657
Diffusion	*Arabidopsis* Donor	F1-score	0.9595	0.1656	0.2356	0.8358	0.9814
		AUROC	0.9899	0.5992	0.7284	0.9512	0.9998
	Arabidopsis Acceptor	F1-score	0.9573	0.2501	0.1737	0.8376	0.9673
		AUROC	0.9904	0.6488	0.6590	0.9370	0.9946
	Human Donor	F1-score	0.9600	0.2272	0.2929	0.9638	0.9791
		AUROC	0.9916	0.6834	0.7289	0.9925	0.9995
	Human Acceptor	F1-score	0.9591	0.1296	0.1485	0.8201	0.9770
		AUROC	0.9890	0.6548	0.5937	0.9527	0.9987

the ability of models trained on synthetic data to generalize to authentic biological sequences. This is a substantially more challenging task than Scenario 1, as it requires synthetic sequences to capture the full distributional complexity of real splice sites rather than merely preserving discriminative motifs.

GAN model performance The GAN model shows poor transferability at low lambda values but gradual improvement with increasing frequency prior strength. At $\lambda = 0.0$, predictive performance is near-zero (F1: 0.0081–0.0329, AUROC: 0.5370–0.6437), indicating that pure GAN-generated sequences fail to provide sufficient training signal for real splice site recognition. At $\lambda = 0.25$, improvement is minimal (F1: 0.0120–0.0598), with predictive performance remaining extremely poor. At $\lambda = 0.5$, modest gains emerge for some datasets (F1: 0.0092–0.2296), though predictive performance remains far below baseline. At $\lambda = 0.75$, more substantial improvement is observed, particularly for *Arabidopsis* donor sites (F1: 0.5433), though other datasets show more modest gains (F1: 0.0156–0.5484). The

highly variable predictive performance across datasets and the overall low F1-scores indicate that GAN-generated sequences, even with strong frequency prior guidance, lack sufficient distributional fidelity for effective training data generation.

VAE model performance The VAE model demonstrates the most consistent transferability, though predictive performance varies across lambda values and datasets. At $\lambda = 0.0$, the VAE model achieves moderate predictive performance (F1: 0.0176–0.4023, AUROC: 0.5842–0.8563), substantially outperforming the GAN and diffusion models at this lambda value. At $\lambda = 0.25$, predictive performance improves notably for several datasets (F1: 0.0496–0.4965), with *Arabidopsis* donor reaching 0.4965. Predictive performance at $\lambda = 0.5$ remains comparable (F1: 0.0406–0.4977), with similar patterns. Interestingly, at $\lambda = 0.75$, predictive performance varies: some datasets show improvement (e.g., *Arabidopsis* acceptor F1: 0.5360) while others decline slightly (e.g., Human acceptor F1: 0.0413). Overall, VAE maintains the most stable and highest transferability across the lambda spectrum, achieving up to 56% of baseline F1-score, though substantial gaps remain compared to training on real data.

Diffusion model performance The diffusion model exhibits highly variable and problematic transferability patterns. At $\lambda = 0.0$, the predictive performance is poor to moderate (F1: 0.1241–0.3390, AUROC: 0.5190–0.7472), exceeding GAN but trailing VAE. At $\lambda = 0.25$, some datasets show improvement (e.g., *Arabidopsis* acceptor F1: 0.4969) while others show modest predictive performance (F1: 0.1013–0.2694). At $\lambda = 0.5$, predictive performance varies widely across datasets (F1: 0.2528–0.6906), with *Arabidopsis* donor achieving the highest transferability (F1: 0.6906, 72% of baseline). However, at $\lambda = 0.75$, a remarkable collapse in predictive performance occurs: several datasets achieve near-zero or zero F1-scores (e.g., *Arabidopsis* donor F1: 0.0000, Human acceptor F1: 0.0072), while AUROC values also decline substantially. This catastrophic failure at high lambda values suggests that the over-concentration on dominant motifs observed in direct evaluation (sequence logos, conservation scores) produces synthetic sequences with insufficient distributional diversity to serve as effective training data.

Transferability insights and lambda trade-offs Scenario 2 results reveal a fundamental trade-off: lambda values that maximize biological realism (Scenario 1) do not necessarily optimize transferability (Scenario 2). For the GAN model, higher λ improves both scenarios, though transferability remains limited. For the VAE model, intermediate lambda values (0.25 and 0.5) provide the best balance, maintaining both realism and transferability. For the diffusion model, a critical threshold emerges: while $\lambda = 0.75$ is essential for Scenario 1 predictive performance, it catastrophically reduces transferability, likely due to over-fitting effects. These results suggest that $\lambda = 0.5$ represents an optimal compromise: it enables strong Scenario 1 predictive performance across all architectures while preserving sufficient distributional diversity for reasonable transferability, particularly for the diffusion and VAE models. Applications requiring synthetic training data should avoid $\lambda = 0.75$ for the diffusion model and carefully evaluate the realism-diversity trade-off when selecting lambda values.

Data Efficiency Analysis

A critical consideration for generative models is their data efficiency, the ability to learn high-quality distributions from limited training sets. This is particularly important for applications involving rare splice variants, non-canonical splicing events, or poorly annotated species where large training datasets may be unavailable. To evaluate data efficiency, we trained each architecture (GAN, VAE, and diffusion) on progressively larger training subsets: 500–10,000 samples for *Arabidopsis thaliana* and 1,000–20,000 samples for *Homo sapiens* splice sites. All models were trained for 50 epochs, and 10,000 synthetic sequences were generated using $\lambda = 0.5$ for each configuration. We assessed both direct sequence characteristics (PWM similarity, `GC` content similarity, and 3-mer context similarity) and indirect functional performance (SpliceRover classification on *Train-Synthetic/Test-Real*).

Table 3: Proxy evaluation results for SpliceRover: *Train-Synthetic/Test-Real*

Model	Dataset	Metric	Real	$\lambda = 0.0$	$\lambda = 0.25$	$\lambda = 0.5$	$\lambda = 0.75$
GAN	*Arabidopsis* Donor	F1-score	0.9595	0.0329	0.0443	0.2296	0.5433
		AUROC	0.9899	0.6437	0.6569	0.7602	0.8618
	Arabidopsis Acceptor	F1-score	0.9573	0.0081	0.0120	0.2093	0.2948
		AUROC	0.9904	0.6421	0.6808	0.7850	0.8154
	Human Donor	F1-score	0.9600	0.0234	0.0598	0.0411	0.0289
		AUROC	0.9916	0.6092	0.6336	0.5855	0.5484
	Human Acceptor	F1-score	0.9591	0.0148	0.0144	0.0092	0.0156
		AUROC	0.9890	0.5370	0.5343	0.5044	0.6219
VAE	*Arabidopsis* Donor	F1-score	0.9595	0.4023	0.4965	0.4221	0.3486
		AUROC	0.9899	0.8367	0.8655	0.8646	0.8918
	Arabidopsis Acceptor	F1-score	0.9573	0.4472	0.3037	0.4977	0.5360
		AUROC	0.9904	0.8563	0.8715	0.8944	0.8872
	Human Donor	F1-score	0.9600	0.0188	0.0496	0.1157	0.0264
		AUROC	0.9916	0.5842	0.5512	0.6514	0.6635
	Human Acceptor	F1-score	0.9591	0.0176	0.0496	0.0406	0.0413
		AUROC	0.9890	0.6641	0.6208	0.6558	0.7255
Diffusion	*Arabidopsis* Donor	F1-score	0.9595	0.3390	0.2694	0.6906	0.0000
		AUROC	0.9899	0.6967	0.7078	0.8849	0.5771
	Arabidopsis Acceptor	F1-score	0.9573	0.4089	0.4909	0.6170	0.0002
		AUROC	0.9904	0.7472	0.7469	0.9062	0.4994
	Human Donor	F1-score	0.9600	0.1334	0.1013	0.2528	0.0700
		AUROC	0.9916	0.5190	0.4892	0.8018	0.6143
	Human Acceptor	F1-score	0.9591	0.1241	0.1171	0.2677	0.0072
		AUROC	0.9890	0.5534	0.5605	0.7354	0.4605

Direct evaluation

Figures 13–16 demonstrate that training set size has minimal impact on the quality of generated sequences. Sequences generated from models trained on small datasets exhibit similarity scores comparable to those from models trained on large datasets.

For PWM similarity, all generated sequences achieve near-perfect scores regardless of training set size, indicating that canonical splice junction motifs are captured equally well with minimal or extensive training data.

GC content similarity shows consistent values across training sizes for all three model types. The observed variation reflects differences across model architectures rather than systematic improvement with increasing training set size.

Similarly, 3-mer context preservation remains stable across all training sizes for each model type. The absence of substantial variation demonstrates that local sequence context is captured effectively even with limited training data.

Indirect evaluation

Figures 17–20 assess functional quality through Train-Synthetic/Test-Real evaluation, where SpliceRover classifier is trained on synthetic sequences, then tested on real sequences.

Unlike direct evaluation metrics, transferability shows considerable variability across training set sizes. Performance fluctuates across different training sizes with no consistent pattern of improvement or degradation.

This variability indicates that while synthetic sequences maintain structural and compositional fidelity (as shown in direct evaluation), their utility as training data for downstream classifiers is less predictable and depends on factors beyond training set size alone.

Computational Complexity Analysis

Training efficiency

Fig. 21 shows average epoch training time for each model. GAN is the fastest, requiring only 0.26 seconds per epoch for *Arabidopsis* and 0.36 seconds for Human, approximately 4 to 7 times faster than VAE and 7 to 10 times faster than diffusion. VAE requires 1.35 seconds (*Arabidopsis*) and 2.55 seconds (Human) per epoch, with the Human training taking nearly twice as long due to the larger training set. The diffusion model is the slowest at 1.84 seconds (*Arabidopsis*) and 3.53 seconds (Human) per epoch, approximately 1.4 times slower than VAE.

Inference efficiency and lambda impact

Fig. 22 presents average inference time per generated sequence (the time it takes to generate a new sequence after the model has already been trained), comparing $\lambda = 0.0$ (*No-Blend*) and $\lambda = 0.5$ (*Blend*) configurations. Frequency blending introduces substantial computational overhead due to position-wise probability distribution computation and linear interpolation with empirical priors. For GAN and VAE, inference at $\lambda = 0.0$ is notably fast, requiring approximately 9 to 10 milliseconds per sequence. Enabling frequency blending ($\lambda = 0.5$) increases inference time roughly 10-fold to 94 to 98 milliseconds per sequence. This overhead is consistent across both species and architectures, indicating that the frequency blending operation itself dominates the additional cost. Despite this increase, GAN and VAE with frequency blending remain fast, generating 10 to 11 sequences per second. The diffusion model shows substantially higher inference costs due to their iterative denoising process. At $\lambda = 0.0$, the diffusion model requires 173 milliseconds per sequence, approximately 17 times slower than GAN/VAE without blending. With frequency blending enabled ($\lambda = 0.5$), inference time increases to 248 to 260 milliseconds per sequence, representing approximately 1.5 times overhead. While the diffusion model remains the slowest architecture with throughput of 4 sequences per second, this computational cost is offset by the superior predictive performance demonstrated in Scenario 1 evaluation at $\lambda \geq 0.5$.

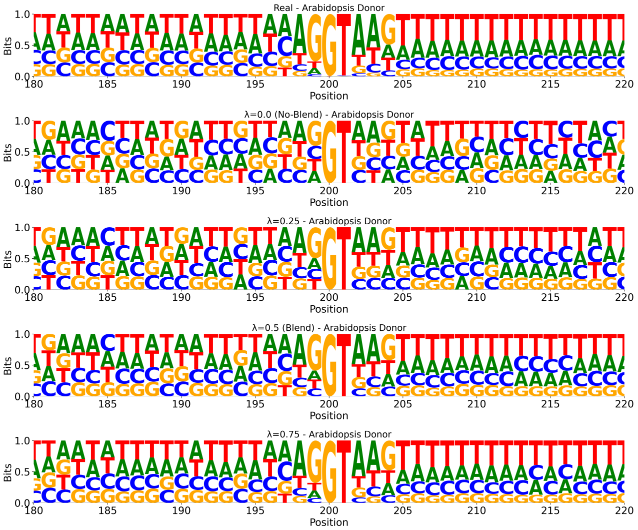

(a) *Arabidopsis* Donor

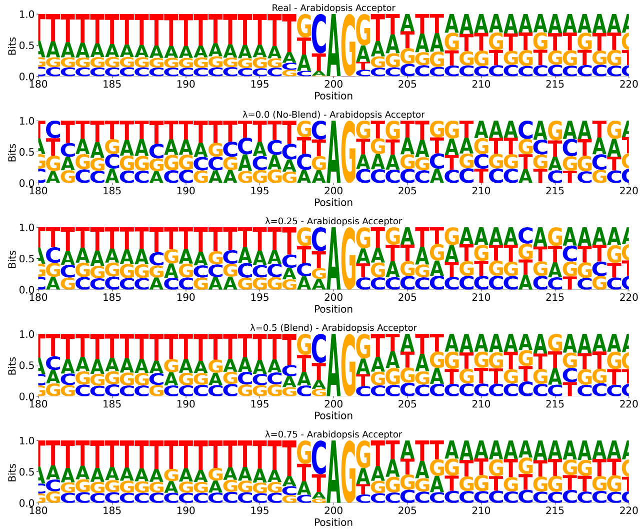

(b) *Arabidopsis* Acceptor

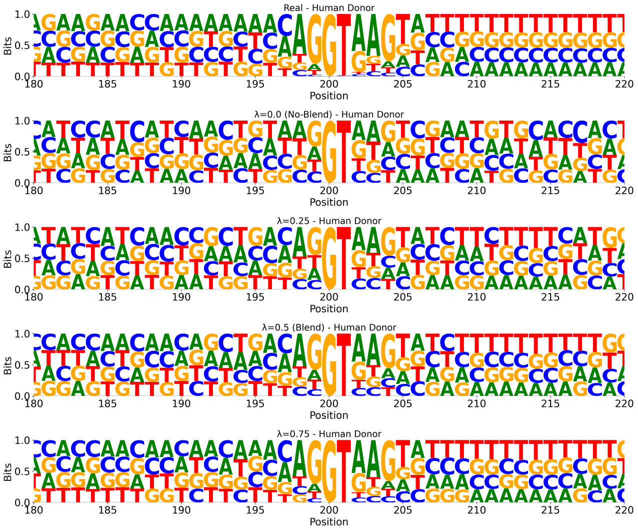

(c) *Homo sapiens* Donor

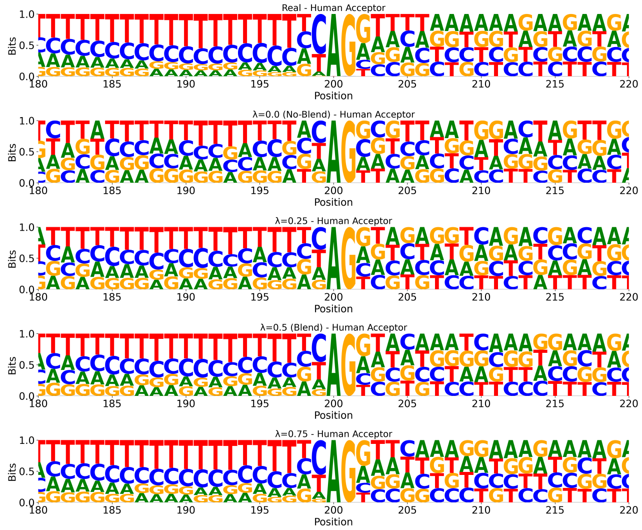

(d) *Homo sapiens* Acceptor

Fig. 10: Comparison of sequence logos for donor and acceptor splice sites: real sequences vs. GAN-generated sequences.

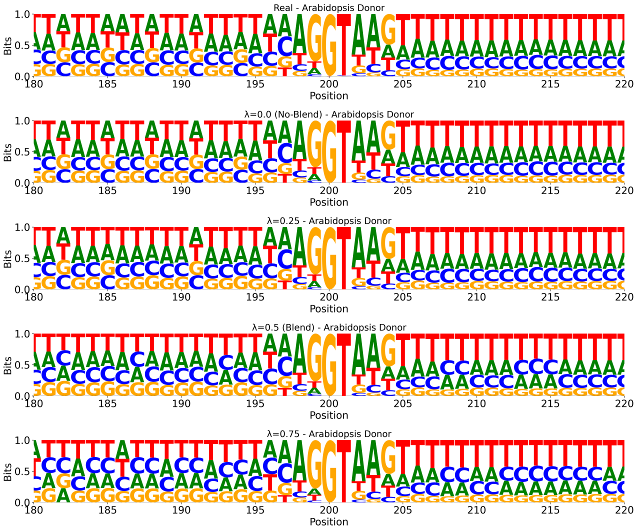

(a) *Arabidopsis* Donor

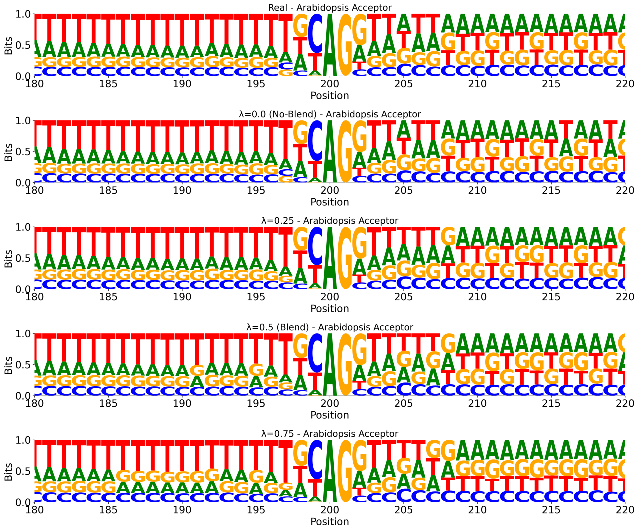

(b) *Arabidopsis* Acceptor

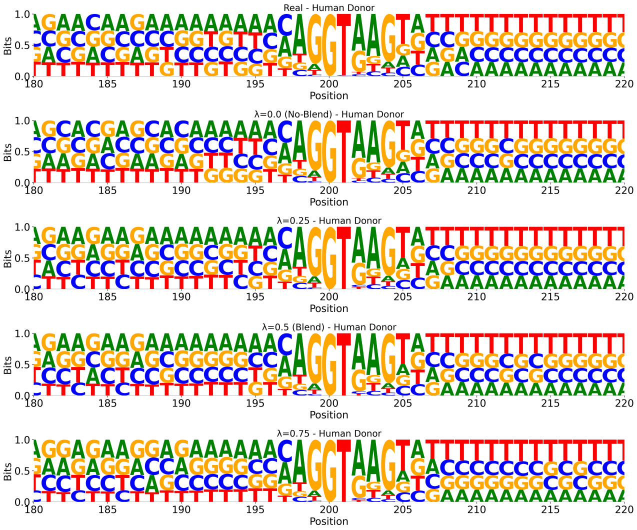

(c) *Homo sapiens* Donor

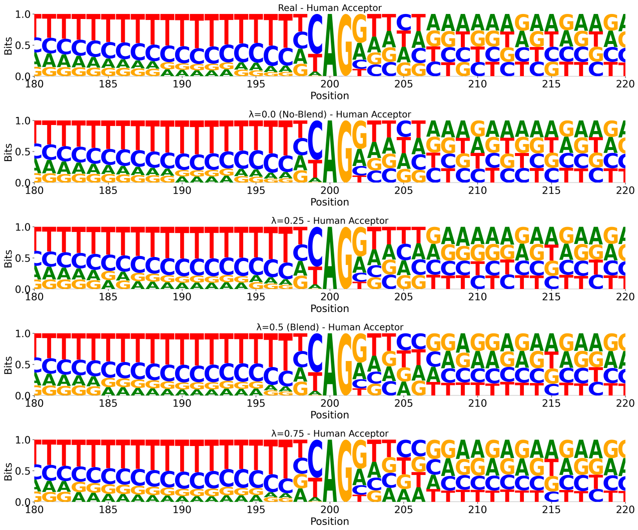

(d) *Homo sapiens* Acceptor

Fig. 11: Comparison of sequence logos for donor and acceptor splice sites real sequences vs. VAE-generated sequences.

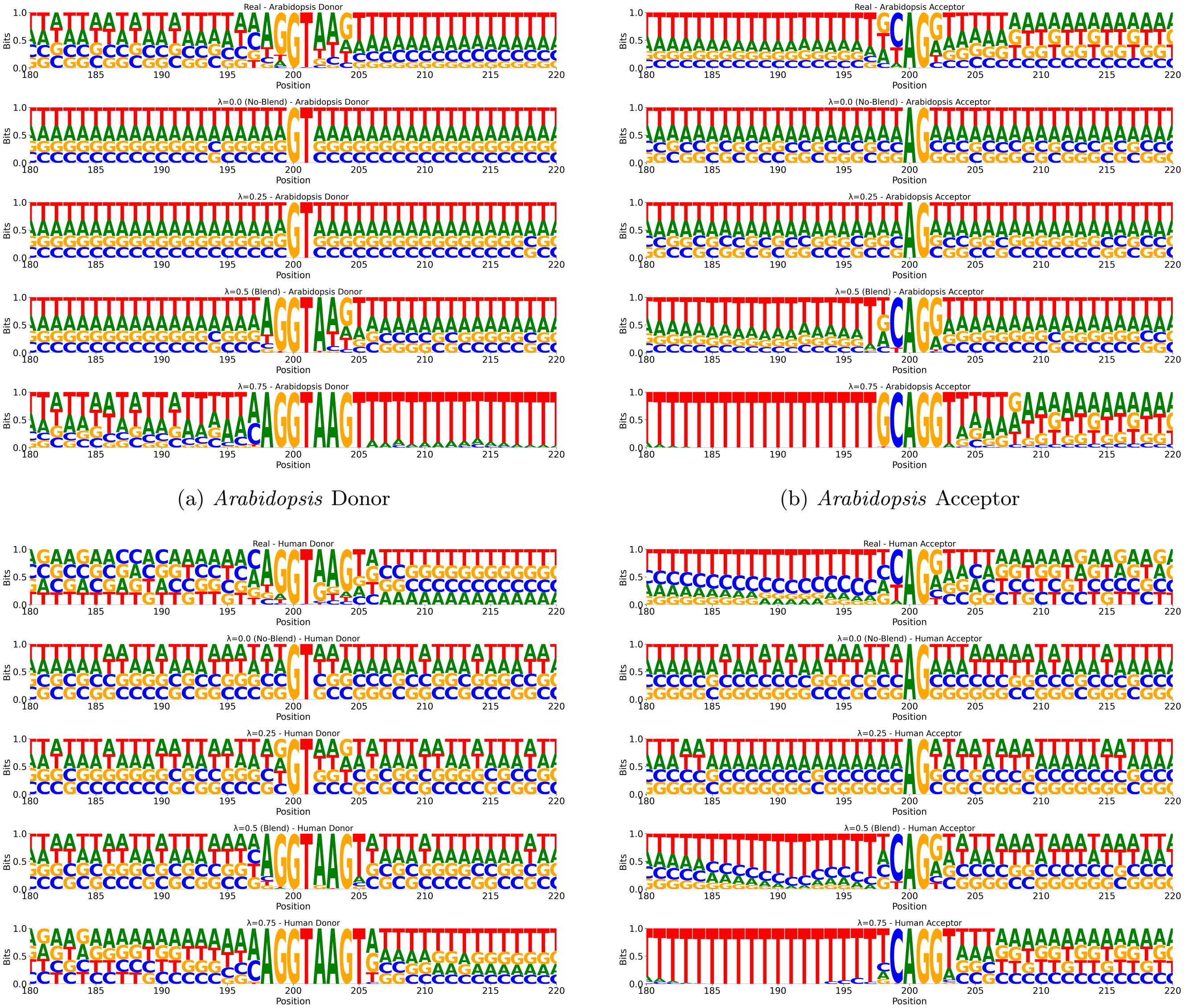

(a) *Arabidopsis* Donor

(b) *Arabidopsis* Acceptor

(c) *Homo sapiens* Donor

(d) *Homo sapiens* Acceptor

Fig. 12: Comparison of sequence logos for donor and acceptor splice sites real sequences vs. diffusion model-generated sequences.

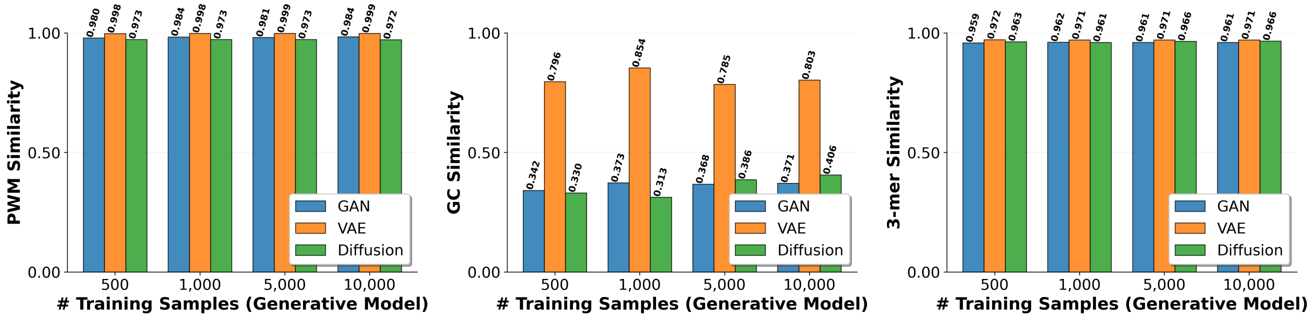

Fig. 13: Direct evaluation metrics for *Arabidopsis* donor splice sites across training set sizes. Left: Position weight matrix (PWM) similarity between generated and real sequences. Center: GC content similarity. Right: 3-mer context similarity.

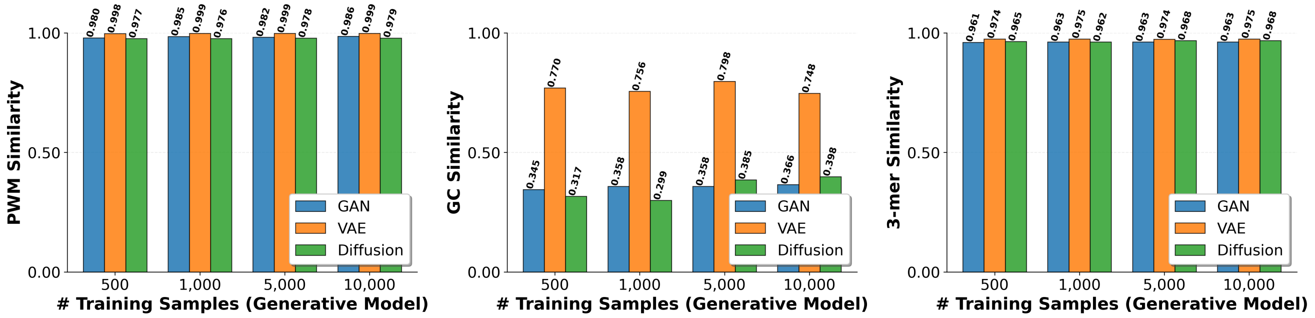

Fig. 14: Direct evaluation metrics for *Arabidopsis* acceptor splice sites across training set sizes. Left: PWM similarity. Center: GC content similarity. Right: 3-mer context similarity.

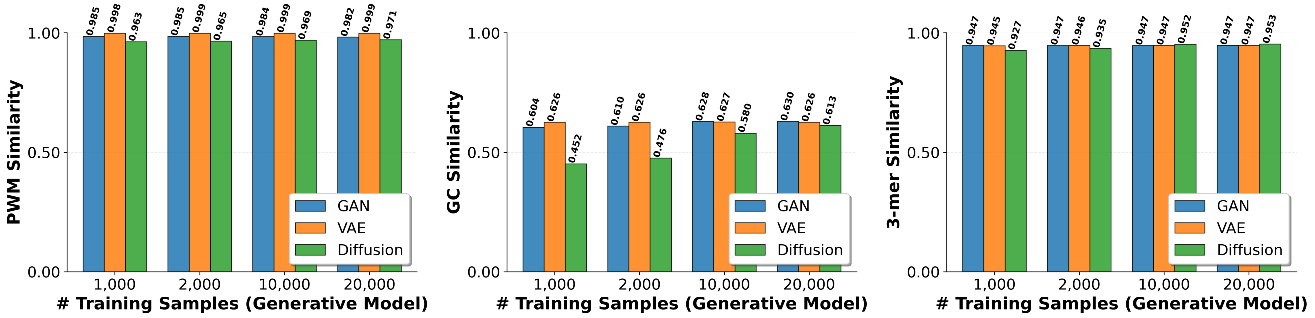

Fig. 15: Direct evaluation metrics for *Homo sapiens* donor splice sites across training set sizes. Left: PWM similarity. Center: GC content similarity. Right: 3-mer context similarity.

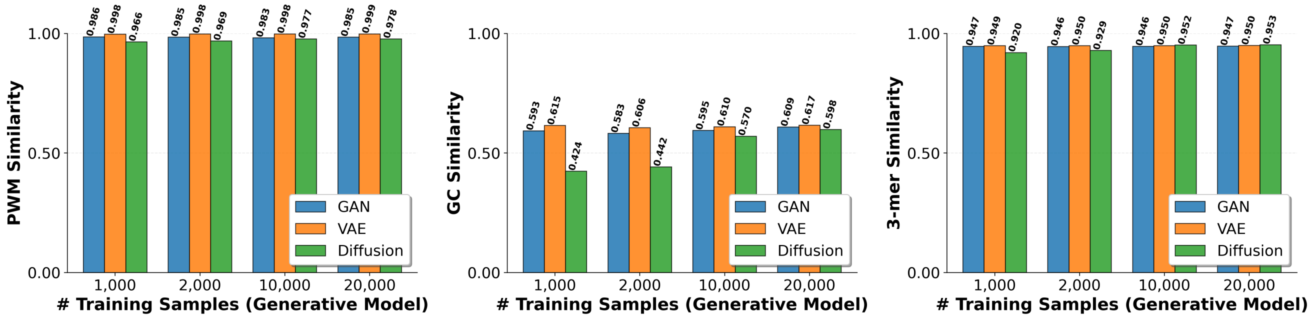

Fig. 16: Direct evaluation metrics for *Homo sapiens* acceptor splice sites across training set sizes. Left: PWM similarity. Center: GC content similarity. Right: 3-mer context similarity.

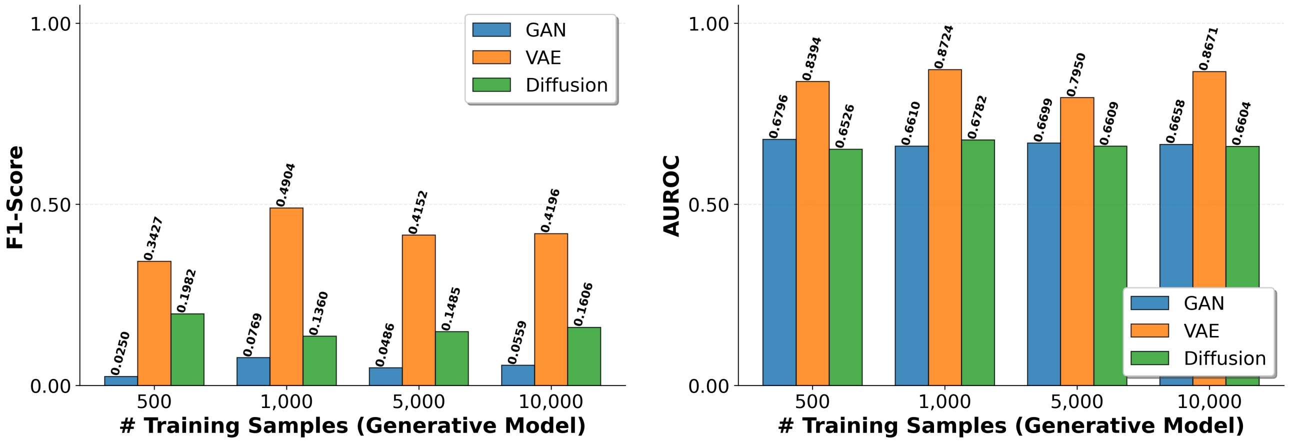

Fig. 17: Indirect evaluation for *Arabidopsis* donor splice sites across training set sizes using SpliceRover (Train-Synthetic/Test-Real). Left: F1-score. Right: AUROC.

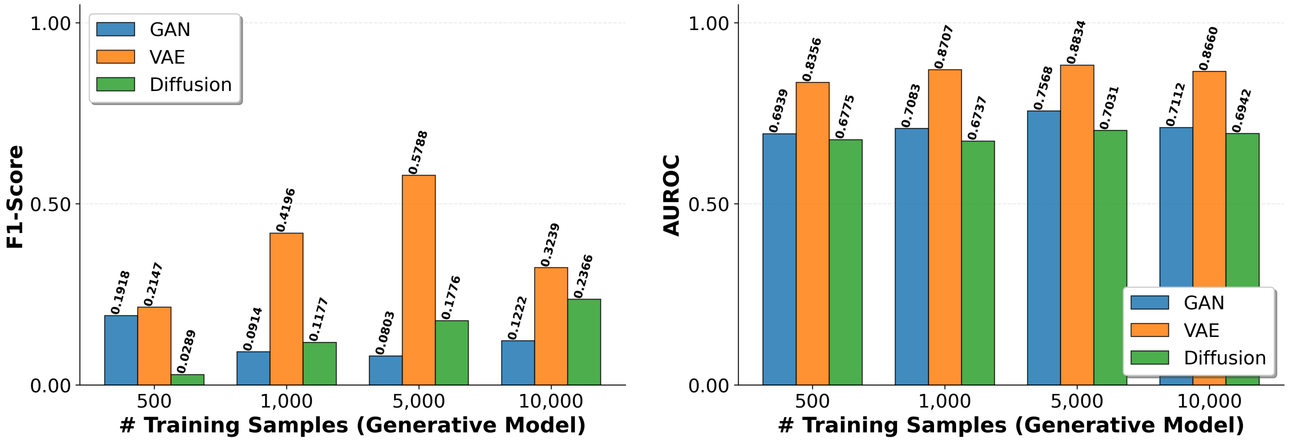

Fig. 18: Indirect evaluation for *Arabidopsis* acceptor splice sites across training set sizes using SpliceRover (Train-Synthetic/Test-Real). Left: F1-score. Right: AUROC.

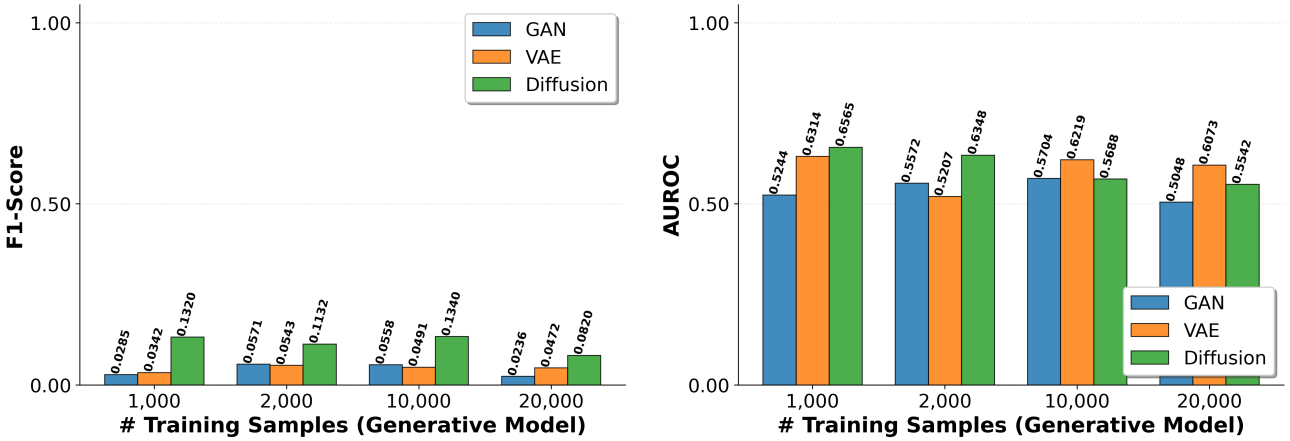

Fig. 19: Indirect evaluation for *Homo sapiens* donor splice sites across training set sizes using SpliceRover (Train-Synthetic/Test-Real). Left: F1-score. Right: AUROC.

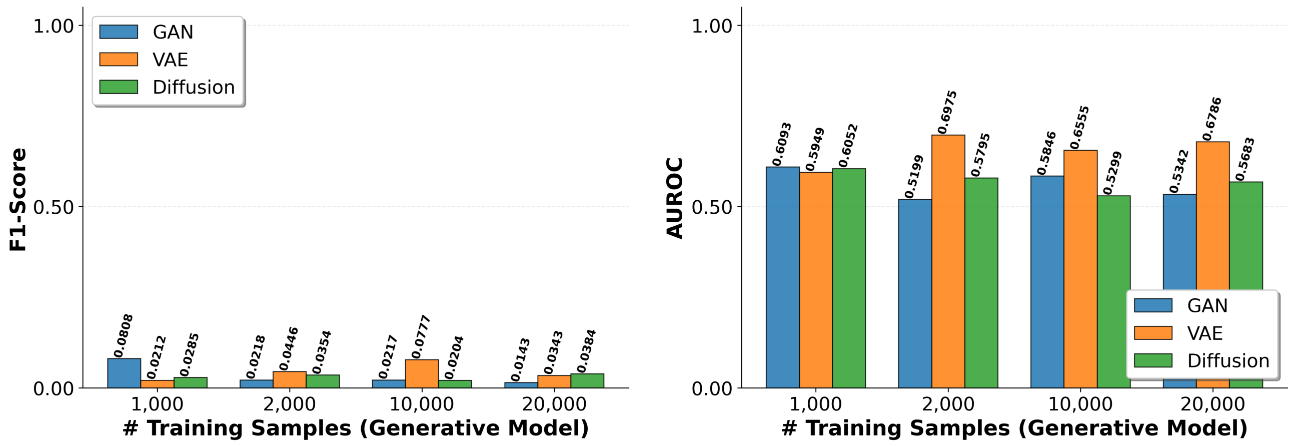

Fig. 20: Indirect evaluation for *Homo sapiens* acceptor splice sites across training set sizes using SpliceRover (Train-Synthetic/Test-Real). Left: F1-score. Right: AUROC.

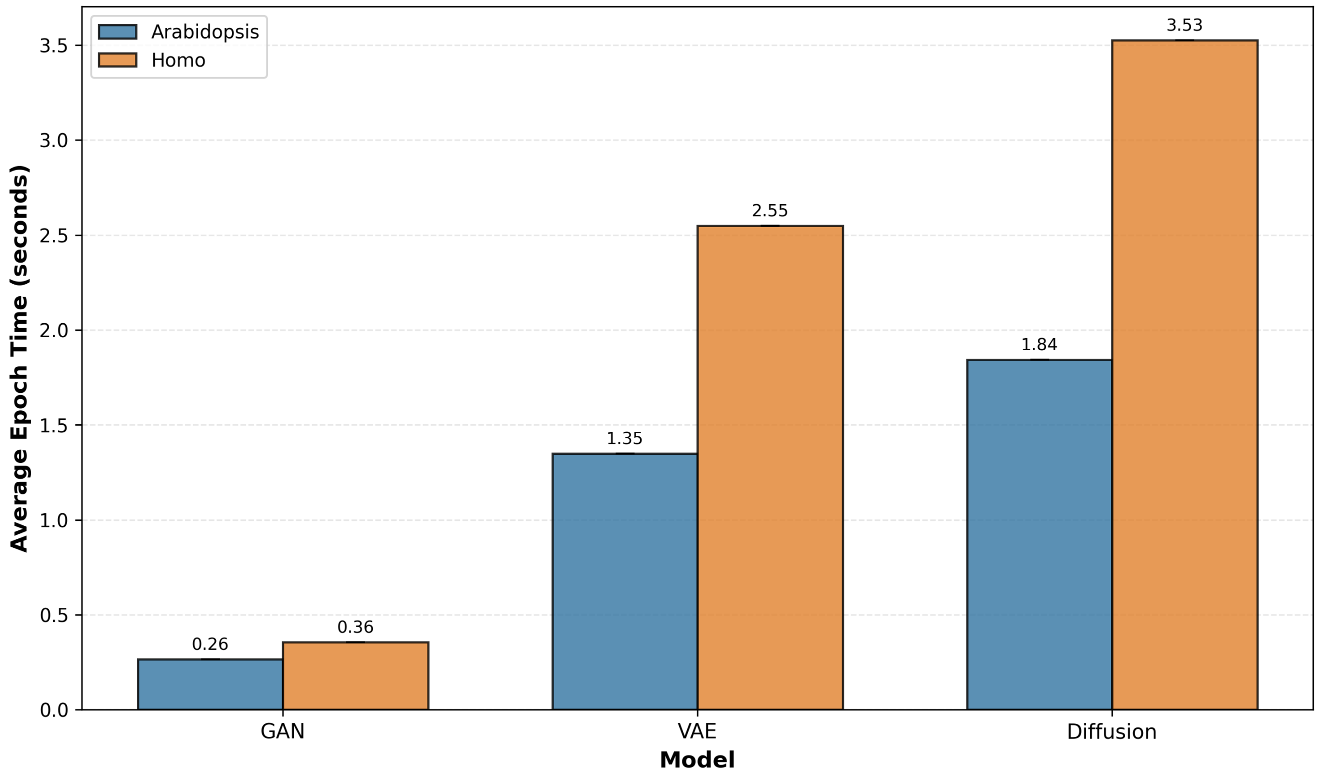

Fig. 21: Average training time per epoch.

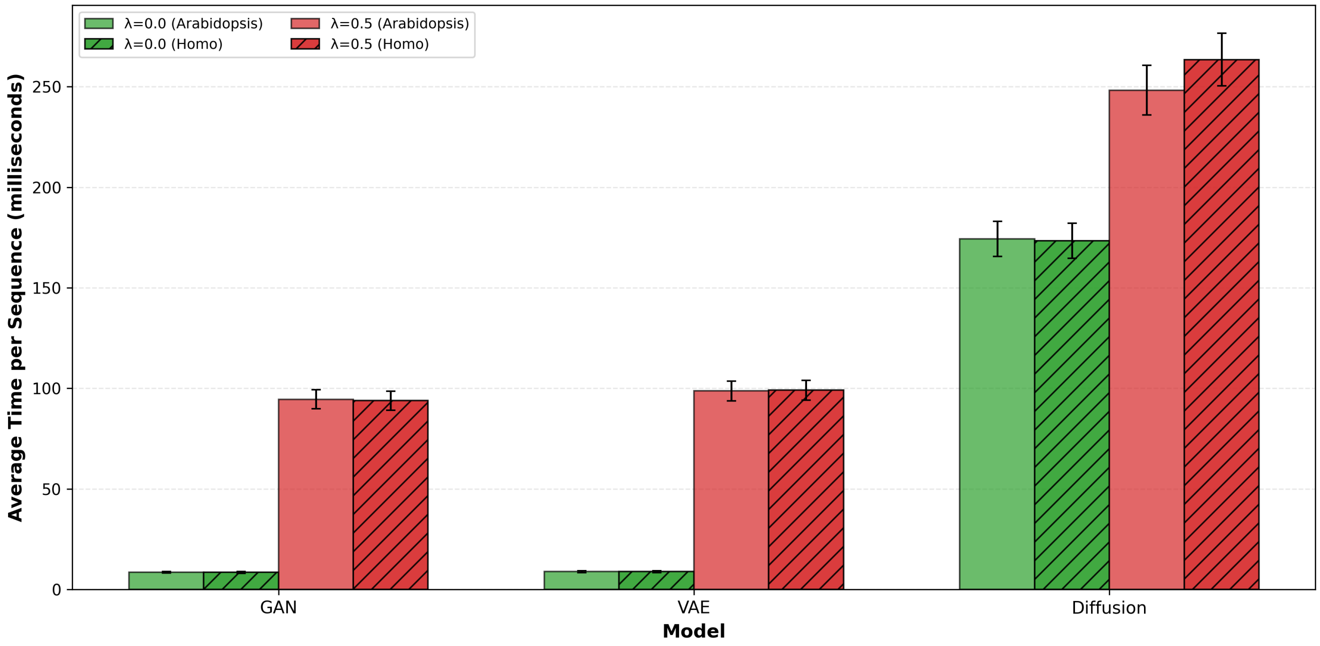

Fig. 22: Average inference time per sequence.

References

1. Zuallaert, J., Godin, F., Kim, M., Soete, A., Saeys, Y., De Neve, W.: SpliceRover: interpretable convolutional neural networks for improved splice site prediction. Bioinformatics 34(24), 4180–4188 (2018)
2. Scalzitti, N., Kress, A., Orhand, R., Weber, T., Moulinier, L., Jeannin-Girardon, A., Collet, P., Poch, O., Thompson, J.D.: Spliceator: multi-species splice site prediction using convolutional neural networks. BMC Bioinformatics 22, 1–26 (2021)
3. Kingma, D.P., Ba, J.: Adam: A method for stochastic optimization. arXiv preprint arXiv:1412.6980 (2014)
